# Discovery of Novel EGFR Inhibitor Targeting Wild-Type and Mutant Forms of EGFR: In Silico and In Vitro Study

**DOI:** 10.3390/molecules28073014

**Published:** 2023-03-28

**Authors:** Duangjai Todsaporn, Alexander Zubenko, Victor Kartsev, Thitinan Aiebchun, Panupong Mahalapbutr, Anthi Petrou, Athina Geronikaki, Liudmila Divaeva, Victoria Chekrisheva, Ilkay Yildiz, Kiattawee Choowongkomon, Thanyada Rungrotmongkol

**Affiliations:** 1 Center of Excellence in Biocatalyst and Sustainable Biotechnology, Department of Biochemistry, Faculty of Science, Chulalongkorn University, Bangkok 10330, Thailand; 2 North-Caucasian Zonal Research Veterinary Institute, 346406 Novocherkassk, Russia; 3 InterBioScreen Ltd., 85355 Moscow, Russia; 4 Department of Biochemistry, Faculty of Science, Kasetsart University, Bangkok 10900, Thailand; 5 Department of Biochemistry, Faculty of Medicine, Khon Kaen University, Khon Kaen 40002, Thailand; 6 Department of Pharmaceutical Chemistry, School of Pharmacy, Aristotle University of Thessaloniki, 54124 Thessaloniki, Greece; 7 Institute of Physical and Organic Chemistry, Southern Federal University, Pr. Stachki 194/2, 344090 Rostov-on-Don, Russia; 8 School of Pharmacy, Ankara University, Ankara 06560, Turkey; 9 Program in Bioinformatics and Computational Biology, Graduate School, Chulalongkorn University, Bangkok 10330, Thailand

**Keywords:** furopyridine, molecular dynamics, drug screening, cytotoxicity, EGFR-TK

## Abstract

Targeting L858R/T790M and L858R/T790M/C797S mutant EGFR is a critical challenge in developing EGFR tyrosine kinase inhibitors to overcome drug resistance in non-small cell lung cancer (NSCLC). The discovery of next-generation EGFR tyrosine kinase inhibitors (TKIs) is therefore necessary. To this end, a series of furopyridine derivatives were evaluated for their EGFR-based inhibition and antiproliferative activities using computational and biological approaches. We found that several compounds derived from virtual screening based on a molecular docking and solvated interaction energy (SIE) method showed the potential to suppress wild-type and mutant EGFR. The most promising **PD13** displayed strong inhibitory activity against wild-type (IC_50_ of 11.64 ± 1.30 nM), L858R/T790M (IC_50_ of 10.51 ± 0.71 nM), which are more significant than known drugs. In addition, PD13 revealed a potent cytotoxic effect on A549 and H1975 cell lines with IC_50_ values of 18.09 ± 1.57 and 33.87 ± 0.86 µM, respectively. The 500-ns MD simulations indicated that **PD13** formed a hydrogen bond with Met793 at the hinge region, thus creating excellent EGFR inhibitory activity. Moreover, the binding of **PD13** in the hinge region of EGFR was the major determining factor in stabilizing the interactions via hydrogen bonds and van der Waals (vdW). Altogether, **PD13** is a promising novel EGFR inhibitor that could be further clinically developed as fourth-generation EGFR-TKIs.

## 1. Introduction

EGFR is a transmembrane protein tyrosine kinase that plays an essential role in cellular signaling for cell proliferation, invasion, metastasis, apoptosis, and angiogenesis [1,2,3,4,5,6]. Structurally, EGFR is composed of three domains—a transmembrane domain, an extracellular domain, and an intracellular tyrosine kinase (TK) domain—which are constructed of four important conserved regions: (i) the activation loop (A-loop), (ii) the glycine-rich loop (G-loop), (iii) the catalytic loop (C-loop), and (iv) the hinge region [7,8]. Deregulation of EGFR is caused by increased EGFR activity such as overexpression and mutations of EGFR [9,10,11]. Increased EGFR activity is associated with numerous malignant tumors, including esophageal cancers, glioblastoma, anal cancers, epithelial cancers of the head and neck, breast cancer, and lung cancers [12,13,14,15,16], especially non-small cell lung cancer (NSCLC). Lung cancer is a major cause of cancer death globally, and NSCLC is the most frequently diagnosed cancer [16,17,18], whereas activating mutations in the tyrosine kinase (TK) domain are recognized as the tumorigenic driver in NSCLC. As a matter of fact, extensive research has been executed to study EGFR function as a therapeutic target for NSCLC.

**Figure 1:**
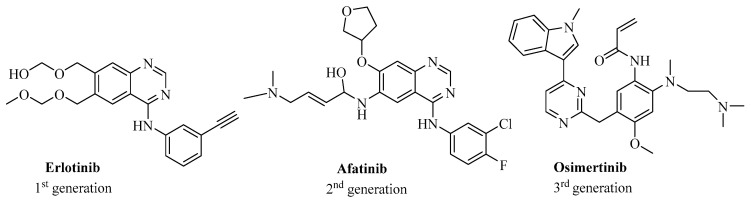
Two-dimensional (2D) structure of three reference drugs targeting EGFR, including erlotinib, afatinib, and osimertinib.

Several EGFR inhibitors have been developed and reported (Figure 1). Erlotinib [19] and gefitinib [20], first-generation reversible EGFR inhibitors, proved to have a strong response and to lead to prolonged survival in NSCLC patients. However, after 9–14 months of treatment, the secondary “gatekeeper” T790M mutation increased ATP-binding affinity and caused recurrence in most NSCLC patients [21]. Second-generation irreversible EGFR inhibitors targeting EGFR with the T790M activating mutation, such as afatinib [22] and dacomitinib [23], were developed to overcome that problem. Although the irreversible covalent bond with C797 confers enhanced sensitivity and selectivity, serious side effects of these TKIs, such as skin rash and diarrhea due to their activity against wild-type EGFR [22,23,24,25], were observed. To combat drug resistance and limit dose-related toxic side effects, Osimertinib (AZD9291), which is a pyrimidine scaffold, was developed as an irreversible third-generation inhibitor [26]. Osimertinib has a high affinity for the drug-resistant L855R/T790M double mutant [27] but no activity against the wild-type EGFR [27,28,29,30]. Additionally, clinical studies revealed that approximately 40% of patients who received osimertinib therapy developed a tertiary mutation (C797S) in the EGFR, resulting in a loss of covalent interactions between the irreversible inhibitors and the side chain of C797 [31]. Therefore, the discovery of potent inhibitors of both wild-type and mutant forms of EGFR is required. Since NSCLC is one of oncology’s “big killers” in the patient population and the EGFR signaling is a main driver for this type of tumor, it is a worthwhile endeavor to develop improved therapeutics in the clinical setting.

Furopyridine (PD, Table 1), a furo[2,3-c]pyridine compound, is a product of a structural modification of vitamin B_6_, which plays an extremely important biological role in living organisms, since it is involved in a wide range of biochemical reactions necessary for cellular metabolism and function. The latter explains why vitamin B_6_ and its derivatives are considered biologically privileged molecules with which to develop new therapeutics.

Furthermore, PDs display a wide range of biological activities, including anticancer [32,33], antiviral [34], anti-inflammatory [35], and HIV protease inhibitory activities [36], and also inhibit α-glucosidase [37]. The products of the furan cyclization of pyridoxal under the action of hetarylamines 2-alkylamino-3-hetarylamino-4-hydroxymethylfuro[2,3-*c*]pyridines have an immunostimulating effect due to their selective activation of Toll-like TLR8 receptors [38]. Even though anticancer activity of **PD** has been reported, an understanding of the binding modes of action of **PD** and its derivatives against NSCLC remains elusive. Hung et al. [33] reported the antiproliferative activity of synthesized thienopyridine derivatives using the panel of NCI-60 cell lines with GI_50_ values in the low nanomolarity range, especially the melanoma cell line MDA-MD-435 (GI_50_—23 nM). Rahman et al. also evaluated the CDK2 inhibitory and antiproliferative activity of pyrazolopyridine, furopyridine, and pyridine derivatives. Furopyridine derivative (14) showed IC_50_ 0.93 µM as a CDK2 inhibitor as well as significant inhibition on different human cancer cell lines (HCT-116, MCF-7, HepG2, and A549).

The main aim of this work was the synthesis and evaluation of novel classes of anti-EGFR agents in order to understand the mechanism of anticancer activity as well as their binding modes to the receptor. Herein, we have reported the synthesis of a number of novel furopyridine derivatives with pharmacologically relevant substituents and also used molecular docking and molecular dynamics (MD) simulations with free energy calculations to virtually screen 14 in-house PD compounds towards wild-type, L858R/T790M, and L858R/T790M/C797S EGFR. Moreover, in vitro EGFR inhibitory and anti-cancer activities against NSCLC cell lines in comparison with normal cell lines were studied. Subsequently, 500-ns MD simulations were carried out to investigate the binding behavior of the most promising PD against wild-type and mutant forms of EGFR. The most promising PD from this work could be further developed as a novel anticancer drug by targeting both wild-type and mutant forms of EGFR.

## 2. Results

### 2.1. Virtual Screening

**Figure 2:**
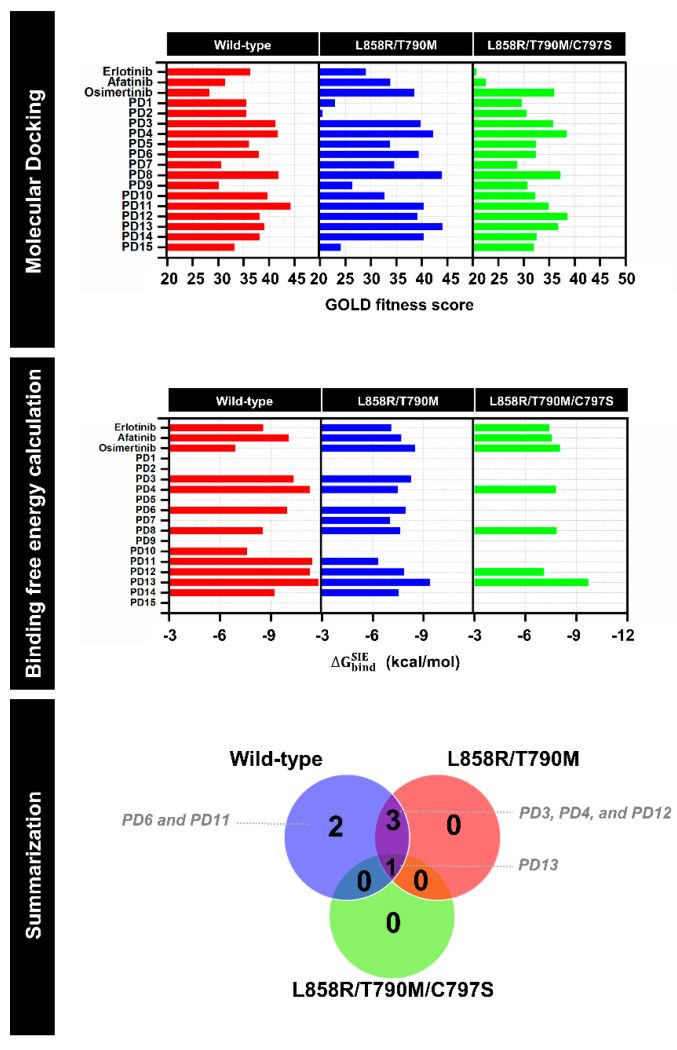
Docking results (**top**), estimated binding free energy calculation based on SIE method (**middle**), and summarization of virtual screening (**bottom**) of furopyridine (**PD**) derivatives towards wild-type, L858R/T790M, L858R/T790M/C797S EGFR relative to three reference drugs (erlotinib, afatinib, and osimertinib).

To assess the potency of EGFR inhibitor(s) against wild-type, L858R/T790M, and L858R/T790M/C797S EGFR, molecular docking was carried out on 14 **PD** compounds. Three drugs—erlotinib, afatinib, and Osimertinib—were selected as a positive control for wild-type, L858R/T790M, and L858R/T790M/C797S EGFR, respectively. The GOLD docking results were plotted in Figure 2. We found that nine **PD** compounds, including **PD3**, **PD4**, **PD6**, **PD8**, and **PD10-PD13**, showed a higher fitness score than erlotinib. Moreover, those **PDs** were found to inhibit L858R/T790M double mutant EGFR. Interestingly, a series of four **PDs** (**PD4**, **PD8**, **PD13**, and **PD14**) exhibited a strong affinity with L858R/T790M/C797S, which is better than osimertinib. The first-round screening suggested that several PD derivatives could be effective in inhibiting both wild-type and mutant forms of EGFR.

To verify the first-round screening, the screened **PDs/**EGFRs complexes derived from molecular docking were subsequently studied on 100 snapshots extracted from the last 10 ns of 100-ns molecular dynamics (MD) simulations. The predicted binding affinity based on the solvated interaction energy (SIE) method of screened **PD** compounds and three positive controls (erlotinib, afatinib, and osimertinib) is shown in Figure 2. **PD6** (ΔG_bind_ of −9.96 kcal/mol) and **PD11** (−11.30 kcal/mol) displayed greater binding affinity than erlotinib (−8.50 kcal/mol) in the wild-type EGFR system, suggesting that both PD6 and PD11 were specific for the wild-type EGFR. Moreover, the three **PDs** (**PD3**, **PD4**, and **PD12**) exhibited the most efficient binding to both wild-type and L858R/T790M EGFR, whereas that against L858R/T790M/C797S EGFR showed poor binding affinities. Surprisingly, the most promising, **PD13**, could be able to suppress both wild-type and mutant EGFR. The ΔG_bind_ calculations of **PD13** based on the SIE method gave ΔG_bind_ of wild-type, L858R/T790M, and L858R/T790M/C797S EGFR as follows: −11.81, −9.39 and −9.7 kcal/mol. Hence, the screened compounds with estimated binding free energies based on the SIE method that were higher than those of the positive controls from each strain of EGFR were synthesized and chosen for in vitro evaluation.

### 2.2. Chemistry

**Scheme 1:**
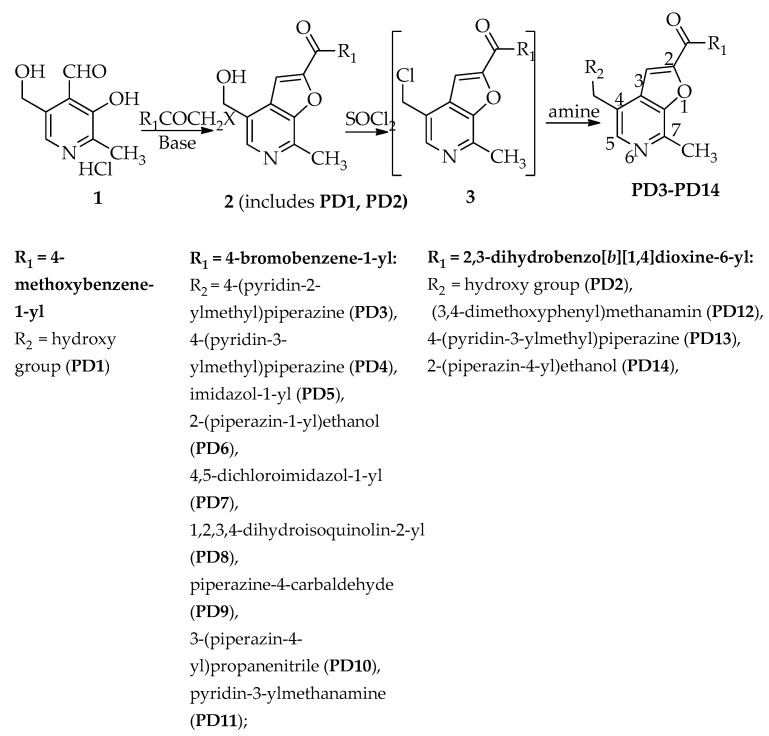
Synthesis of target compounds.

All the target compounds were synthesized as outlined in Scheme 1.

The cyclization of pyridoxal hydrochloride **1** in aqueous acetonitrile at 50–60 °C used potassium carbonate as a catalyst soft base, and proceeded fairly quickly in all cases—within 2–3 h—and led to the synthesis of 4-hydroxymethyl-7-methylfuro[2,3-*c*]pyridines (**PD1**, **PD2**). The yields of compounds **PD1**, **PD2** were 55% and 79%, respectively.

The replacement of a hydroxyl group by a chlorine atom in compounds **PD1, PD2**, by the action of thionyl chloride in DMF furnished the target compounds **2** [39]. Further, the reaction of the resulting chloromethyl derivatives **3** with alkyl-and dialkylamines resulted in the formation of the corresponding 4-aminomethylfuro[2,3-*c*]pyridines **PD3-PD14**. It was more convenient to carry out the reactions in EtOH (for **PD3**-**PD6**, **PD9**-**PD11**, 25 °C—reflux, 3–6 h) or DMF (for **PD7**, **PD8**, **PD12**, **PD13**, 30–40 °C, 3–5 h).

The R_1_ and R_2_ substituents were chosen considering that the biological activity of the compounds, as well as their pharmacodynamics and pharmacokinetics, are significantly affected by both electronic factors (electron donation or electron acceptor of substituents) and their physical properties (lipophilicity, hydrophilicity, polarizability, etc.)

The structure of the obtained furo[2,3-*c*]pyridines was confirmed by ^1^H NMR spectroscopy. ^1^H NMR data for compounds **PD1**, **PD2** can be illustrated by the example of 4-hydroxymethylfuro[2,3-*c*]pyridines **PD1**. The ^1^H NMR of this ketone showed the proton signals of H-3 and H-5 of the bicyclic system (singlets at 7.79 and 8.24 ppm, respectively), the benzoyl group (two-proton, somewhat distorted doublet at 8.10 ppm (H-2′, H-6′, *J* 8.7 Hz) as well as multiplate at 7.08–7.14 (2H, H-3′, H-5′)), the hydroxymethyl group (two-proton and single-proton singlets at 4.79 and 5.24 ppm, respectively), and the methyl group (singlet at 2.76 ppm) (see Supplementary Files, ^1^H-NMR spectra) (SI).

In the spectra of amino derivatives **PD3**-**PD14**, there was no signal of the CH_2_OH group, while in the hydrochlorides of these compounds (**PD3**-**PD5**, **PD9**, **PD11**-**PD14**), a broadened singlet of the NH^+^ group appeared in the region of 5.01–5.71 ppm. The aromatic proton signals of these compounds (**PD3**-**PD14**) correspond to the proposed structures and differ from the signals of 4-hydroxymethylfuro[2,3-c]pyridine (**PD1**) by 01–03 ppm (see Supplementary Files, ^1^H-NMR spectra). The ^13^C-NMR of all these compounds was in agreement with their structure (see Supplementary Materials, ^13^C-NMR).

### 2.3. Kinase Inhibitory Activities of EGFR

**Figure 3:**
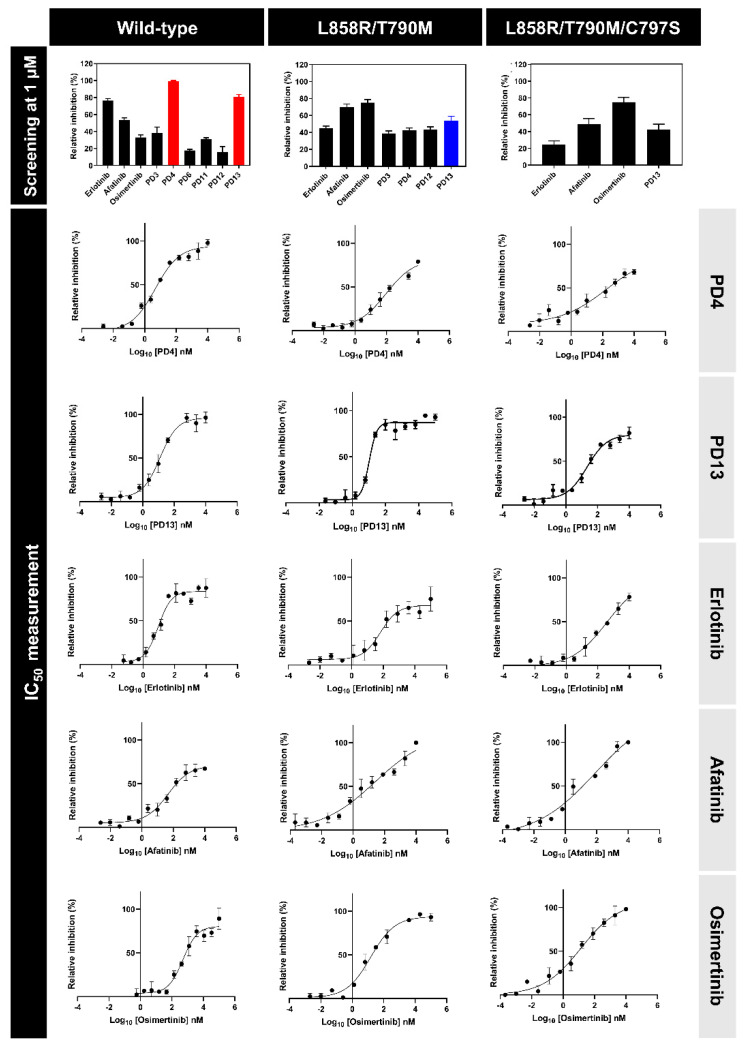
Three forms of EGFR (wild-type, L858R/T790M, and L858R/T790M/C797S) inhibitory screening of PD derivatives at 1 µM concentration (**top**) and IC_50_ curves of compounds that present more than 50% **PD4** and **PD13** in comparison with reference drugs (**bottom**).

Potent compounds obtained from in silico screening were selected to elucidate their inhibitory EGFR activity against different types of EGFR (wild-type, L858R/T790M and L858R/T790M/C797S). Note that erlotinib, afatinib, and osimertinib, which are FDA-approved drugs targeting EGFR, were used as the positive control. The selected compounds and drugs were first screened to observe the sign of EGFR using a single 1 µM dose concentration. The **PD** compounds that present a percentage value of EGFRs inhibition <50 were selected to calculate the IC_50_ values. The relative inhibition of EGFR and IC_50_ curves were analyzed and are plotted in Figure 3, and the IC_50_ values of focused **PD** and drugs are summarized in Table 2. It was found that **PD13** was active in wild-type and L858R/T790M forms of EGFR, whereas **PD4** was only against wild-type EGFR.

The IC_50_ values of **PD4** and **PD13** were determined in comparison with reference drugs (Table 2). The IC_50_ values of two **PD** compounds and drugs inhibiting three strains of EGFR were in the nanomolar range. **PD4**/wild-type (IC_50_ of 4.78 ± 0.73 nM) displayed EGFR inhibitory activity >2-fold compared to erlotinib (of 14.11 ± 0.19 nM). On the other hand, **PD4** complexed with L858R/T790M (91.02 ± 2.01 nM) and L858R/T790M/C797S EGFR (103.70 ± 3.62 nM) gave the IC_50_ value of >100 nM, suggesting that **PD4** is specific against wild-type EGFR. **PD13** showed the lowest IC_50_ values for both wild-type (11.64 ± 1.30 nM) and L858R/T790M (10.51 ± 0.71 nM). While **PD13** inhibited L858R/T790M/C797S EGFR with IC_50_ values in the double-digit nanomolar range, it had lower potencies than third-generation EGFR inhibitor osimertinib. Furthermore, the **PD13**/triple mutant EGFR appeared to be a more promising candidate compound than previously reported L858R/T790M/C797S EGFR inhibitors such as 2,9-disubstituted-8-phenylthio/phenyl-sulfinyl-9H-purines (IC_50_ of 114 nM) [40], quinoline derivative (IC_50_ of 113 nM) [41], or 5-methylpyrimidopyridone derivative (IC_50_ of 27.5 nM) [42].

### 2.4. Antiproliferative Activities on NSCLC and Normal Cell Lines

The antiproliferative activity of two focused **PD** compounds and drugs were evaluated against NSCLC cell lines, namely the wild-type NSCLC cell line A549 as well as L858R/T790M mutant NSCLC cell line H1975. Erlotinib, afatinib, and osimertinib were used as positive controls. The results are summarized in Table 3. We found that both **PD4** and **PD13** exhibited strong anti-cancer activity by inhibiting the proliferation of A549 rather than H1975 cell lines. In addition, **PD13** exhibited stronger antiproliferative activities than afatinib and osimertinib against the A549 cell line. Moreover, **PD13** showed cell growth inhibition in H1975 cell cultures at a similar level to afatinib.

Furthermore, **PD4, PD13**, and positive controls were then investigated for their cytotoxicity to the normal cell line Vero (monkey kidney epithelial cells). We found that the IC_50_ value of **PD13** was >100 µM in Vero cells, whereas for **PD4** IC_50_ was <100 µM. These results suggested that **PD13** showed low cytotoxicity against the normal cell. Therefore, **PD13** was chosen in order to study its binding affinity by MD simulations.

### 2.5. System Stability

**Figure 4:**
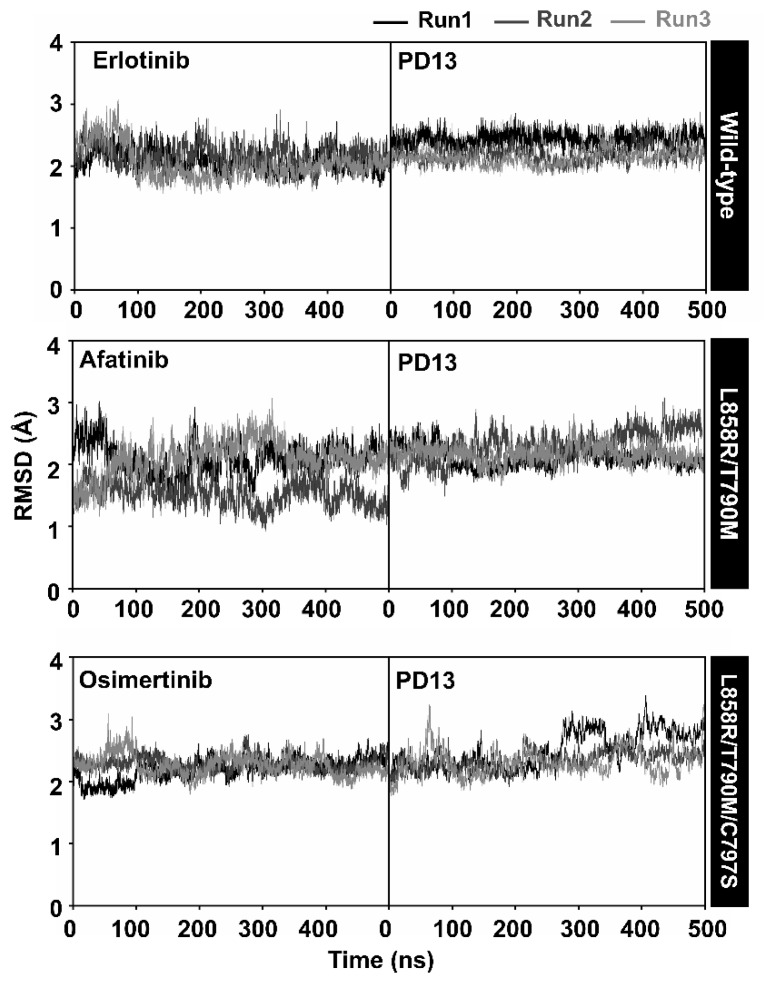
All-atom RMSD of EGFRs with **PD13** and drugs during 500 ns of simulation from three independent simulations.

The stability of **PD13** and the reference drugs erlotinib, afatinib, and osimertinib was determined using all-atom root mean square displacement (RMSD) to observe the system stability. The RMSD values of each system were obtained from three independent simulations. As shown in Figure 4, the RMSD values of the erlotinib system dramatically increased in the first 100 ns and were maintained at the fluctuation of ~2.0–3.0 Å until the end of the simulation for all independent runs. The wild-type/**PD13** complexes reached three runs after 300 ns. The RMSD values of afatinib complexed with L858R/T790M EGFR fluctuated ~1.5–3.0 Å and were maintained at the fluctuation of ~2.0–3.0 Å until the end of the simulation. In the case of **PD13/**double mutant, EGFR showed similar patterns to the three independent simulations. The RMSD value of osimertinib continuously increased in the first 100 ns and was stably maintained at a fluctuation of 3.0–4.0 Å. L858R/T790M/C797S complexed with **PD13** appeared to be quite stable after 400 ns for all simulated systems, while the RMSD value of **PD13** and known drugs complexed with EGFRs of all systems tended to be stable after 200 ns. Altogether, the MD trajectories from 400 to 500 ns of each simulation were extracted for further analysis in terms of: (i) key binding residues; (ii) protein-ligand hydrogen bonding; and (iii) binding affinity.

### 2.6. Key Binding Residues

**Figure 5:**
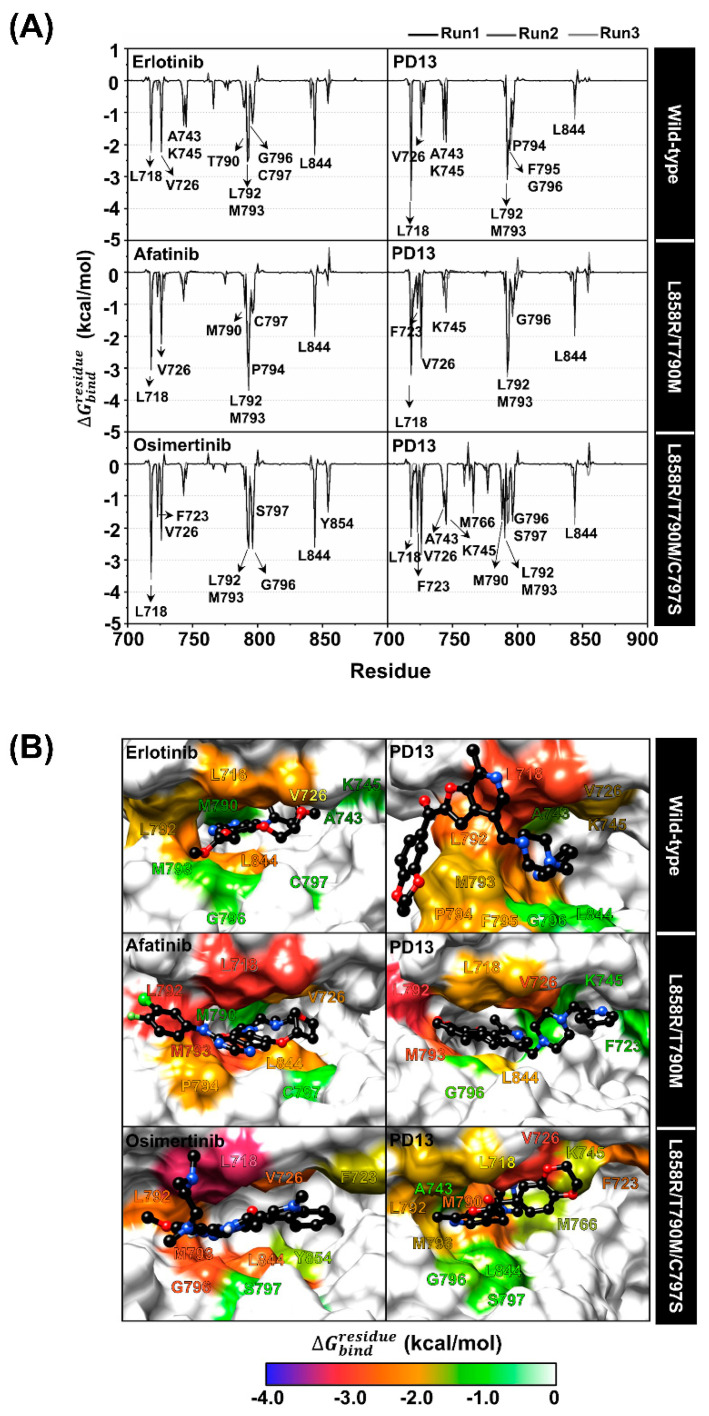
(**A**) Per-residue decomposition free energy of EGFRs for the binding of **PD13** and drugs from three independent simulations (**top**). (**B**) The binding orientation of **PD13** and drugs within the binding pocket of EGFR was drawn from the last snapshot. The lowest and highest energies are shaded from blue to white (**bottom**).

To explore the binding affinity of **PD13** and reference drugs, the per-residue free energy decomposition ($\Delta G_{\mathrm{bind}}^{\mathrm{residue}}$) based on the MM/PBSA method was calculated on 100 snapshots extracted from the last 100 ns of all simulations. Note that, among the 695–1018 residues of EGFR, only the energy stabilization of ≤−1.0 kcal/mol for the 700–900 residues of all systems was plotted in Figure 5. The compounds were shaded based on their highest and lowest energies, with white representing the highest energies and blue representing the lowest energies.

The wild-type/erlotinib showed important residue for binding ten residues including L718, V726, A743, K745, T790, L792, M793, G796, C797, and L844. Interestingly, the PD13/wild-type complex displayed a similar binding pattern to that of reported EGFR inhibitors and was stabilized by L718 (−3.74 kcal/mol, dark red) at the hinge region, which is in good agreement with a previous study that identified L718, A743, L792, M793, G796, and L844 as key residues involved in the main binding of EGFR inhibitors [43,44,45]. In addition, the residue M793 of L858R/T790M EGFR exhibited the highest stabilization for **PD13** (−3.43 kcal/mol, dark red) compared to the afatinib (−3.21 kcal/mol, red) system. This may be one of the reasons that explains why **PD13** could inhibit L858R/T790M EGFR better than afatinib. The binding of PD13 with L858R/T790M/C797S EGFR displayed a higher number of amino acid residues than Osimertinib; L718, V726, A743, K745, M766, L788, M790, L792, M793, G796, S797, and L844 are all involved in ligand stabilization. Among all the residues, the moderate L858R/T790M/C797S EGFR inhibitory potency of **PD13** may be due to steric clashing of M790 with the phenyl ring of the inhibitor.

### 2.7. Protein-Ligand Hydrogen Bonding

**Figure 6:**
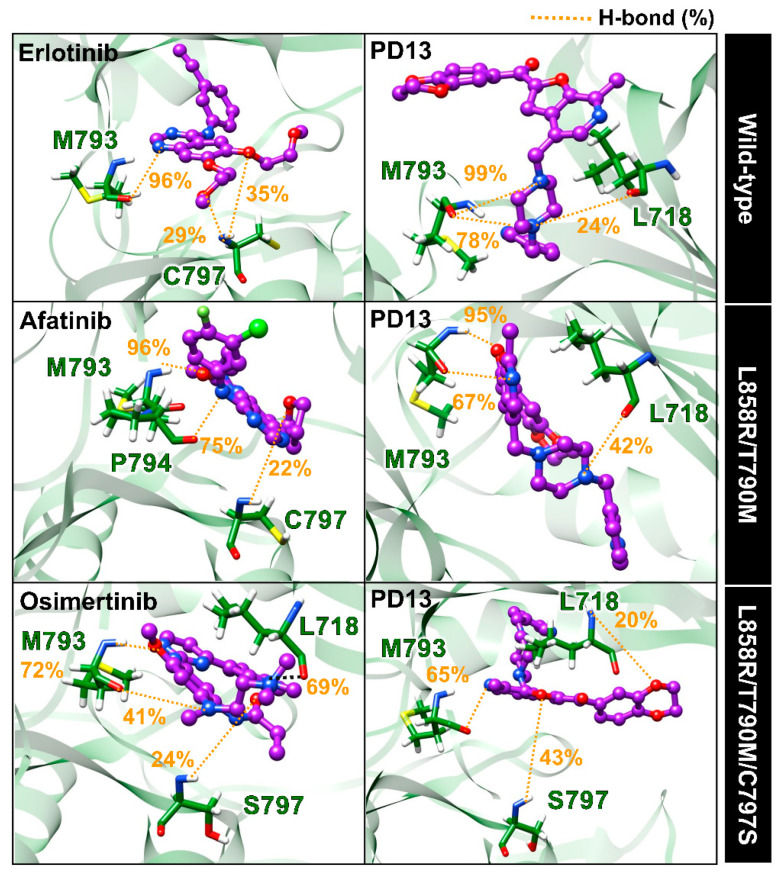
The three-dimensional representative structures showing a percentage of the H-bond occupations of **PD13** and drugs with EGFRs, drawn from the last snapshot of run1. The yellow dashed lines indicate H-bond formation.

The hydrogen bond is one of the main factors for determining the binding strength of the protein–ligand complex. We further calculated the hydrogen bond occupations between EGFRs with **PD13** and reference drugs using the two following criteria: (i) distance between hydrogen donor (HD) and acceptor (HA) ≤ 3.5 Å; and (ii) the angle between HD and HA being over 120° (HD−H⋯HA ≥ 120°). The percentages of hydrogen bond occupations for the studied ligands were observed and are compared in Figure 6.

The hydrogen bond formations of wild-type/**PD13** indicated strong stabilization with the M793 (78–98%) and L718 (24%), and are similar to that found in wild-type/erlotinib complex. In the case of L858R/T790M double EGFR, this compound formed a hydrogen bond with M793 (67–95%) and L178 (42%), in good agreement with the per-residue decomposition analysis mentioned above. **PD13** complexed with triple mutant EGFR showed similar H-bond formations to those of other forms of EGFR (except S797). We speculate that S797 residue that is mutated EGFR could lead to moderate inhibitory activity of L858R/T790M/C797S EGFR due to more favorable interactions with S797 via a hydrogen bond. Therefore, the inhibitory activity of **PD13**/EGFRs was induced by the two key residues, which are M793 and S797, at the hinge region. The main EGFR residues (L718, M793, and S797) evidenced crucial binding via hydrogen bonds, which is similar to other reported EGFR tyrosine kinases such as tupichinol E [46], quinazoline [47], and 9-heterocyclyl substituted 9H-purine derivatives [48].

### 2.8. Binding Affinity

The binding efficiency of **PD13** and reference drugs was calculated using the MM/PBSA approach on 100 snapshots extracted from the last 100 ns of three independent simulations. The values of the averaged ∆G_bind_ over the three independent simulations are listed in Table 4 in comparison with the half-maximal inhibitory concentration (IC_50_).

In the gas term, we found that vdW interactions were three- to fourfold higher than electrostatic interactions in all studied ligands. Therefore, vdW interactions play an important role in the recognition of **PD13** and reference drugs. These interaction findings are strongly consistent with previous studies on several small-molecule therapeutic kinase inhibitors such as olmutinib, lapatinib, and icotinib [49,50,51]. From the ∆G_bind_ calculation, the predicted binding values produced similar phenomena to that of experimental ∆G_bind_ values converted from the IC_50_ values, indicating that the MM/PBSA approach produced reliable ∆G_bind_ results. It was found that **PD13** complexed with the wild-type and L858R/T790M system had a better binding affinity than erlotinib or afatinib, respectively. While **PD13** complexed with L858R/T790M/C797S displayed lower binding affinity than osimertinib, the results agree well with in vitro L858R/T790M/C797S EGFR inhibitory activities, showing that this compound could inhibit wild-type and L858R/T790M better than L858R/T790M/C797S EGFR.

## 3. Materials and Methods

### 3.1. General Information

NMR ^1^H (300 MHz) and ^13^C (125 MHz) spectra of newly synthesized compounds were recorded on a spectrometer Bruker AC-300 (300 MHz) and BRUKER AVANCE NEO 500 MHz FT spectrometers in DMSO-*d*6. Chemical shifts of nuclei ^1^H were measured relative to the residual signals of deuterosolvent (δ = 2.50 ppm). Coupling constants (*J*) are reported in Hz. Melting points were determined by using Fisher-Johns Melting Point Apparatus (Fisher Scientific) and are uncorrected. Elemental analysis was performed using the classical method of microanalysis. The reaction and purity of the obtained compounds were monitored by TLC (plates with Al_2_O_3_ III activity grade, eluent CHCl_3_, development of TLC plates by exposition to iodine vapors in “iodine chamber”).

### 3.2. Chemistry

The reaction conditions and yields of compounds **PD3–PD14** are presented in Table 5.

Compound **PD2** was synthesized as described previously [39].

*[4-Hydroxymethyl-7-methylfuro[2,3-c]pyridin-2-yl](4-methoxyphenyl)methanone* (**PD1**).

The mixture of pyridoxal hydrochloride **1** (1.02 g, 0.05 mol) and 2-bromo-1-(4-methoxyphenyl)ethan-1-one (1.0 g, 0.05 mol) with a saturated aqueous solution of K_2_CO_3_ (10 mL), CH_3_CN (10 mL) and 0.05 g TEBA was stirred vigorously at 20–25 °C for 1 h and at 50–60 °C for 3 h. The mixture was cooled, water (50 mL) was added, and the formed precipitate was filtered off and washed with water (15 mL). Yield: 0.76 g (57%). Light beige crystals, m.p. 147–148 °C (CH_3_CN). ^1^H NMR (DMSO-*d*6: δ ppm 2.76 (s, 3H, Me), 3.93 (s, 3H, OCH_3_), 4.79 (s, 2H, CH_2_), 5.24 (br. s, 1H, OH), 7.08–7.14 (m, 2H, H-3′, H-5′), 7.79 (s, 1H, H-3), 8.10 (d, 2H, *J* 8.7, H-2′, H-6′), 8.24 (s, 1H, H-5)). ^13^C NMR (DMSO-*d*6) δ ppm: 18.322, 55.607, 58.928, 113.455, 114.154, 128.664, 129.946, 130.748, 131.870, 139.910, 142.353, 150.118, 152.76, 163.576, 181.882 (DMSO-*d*6: 38.795–40.045). Anal.Calc. for C_17_H_15_NO_4_; (%). C, 68.68; H, 5.09; N, 4.71%. Found: C, 68.53; H, 5.00; N, 4.67%.

#### General Procedure for the Synthesis of 7-Methylfuro[2,3-c]pyridine Derivatives **PD3–PD14**

A mixture of corresponding 4-chloromethyl derivative **3** (3 mmol) [41], saturated K_2_CO_3_ solution (3 mL) and corresponding amine (3 mmol) in solvent was stirred. Conditions for the reaction are given in Table 1. Then, 50 mL of water was added, and the formed precipitate was filtered off and washed with water (15 mL). To convert the amines **PD3–PD5**, **PD9**, and **PD11–PD14** into their hydrochlorides, they were treated with a saturated solution of HCl in isopropanol until a weakly acidic reaction occurred (pH~3).

*(4-Bromophenyl){7-methyl-4-[(4-(pyridin-2-ylmethyl)piperazin-1-yl]methyl}furo[2,3-c]pyridin-2-yl)methanone dihydrochloride* (**PD3**).

Yield: 1.08 g (62%). Light beige crystals, m.p. 224–225 °C (EtOH). ^1^H NMR (DMSO-*d*6): δ ppm 2.98 (s, 3H, CH_3_), 3.48 (s, 4H, H-3′, H-5′), 3.60 (s, 4H, H-2′, H-6′), 4.45 (s, 2H, CH_2_), 4.84 (s, 2H, CH_2_), 6.54 (br. s, 2H, 2NH^+^), 7.59–7.65 (m, 1H, H-3), 7.79–7.82 (m, 2H, H-3‴, H-5‴), 7.91 (t, *J* 7.8, 1H, H-4‴), 8.11–8.15 (m, 3H, H-2″, H-6″, H-6‴), 8.60 (s, 1H, H-3″), 8.70–7.72 (m, H-5″), 8.85 (s, 1H, H-5). ^13^C NMR (DMSO-*d*6) δ ppm: 15.936, 47.989, 52.125, 57.351, 115.179, 125.002, 126.285, 128.292, 131.703, 132.094, 134.511, 137.749, 140.949, 143.629, 146.405, 149.727, 150.445, 155.023, 182.261 (DMSO-*d*6: 38.872–40.128) Anal.Calc. for C_26_H_27_BrCl_2_N_4_O_2_;(%): C, 54.00; H, 4.71; Br, 13.82; Cl, 12.26; N, 9.69%. Found: C, 54.11; H, 4.62; Br+Cl, 26.21; N, 9.57%.

*(4-Bromophenyl){7-methyl-4-[(4-(pyridin-3-ylmethyl)piperazin-1-yl]methyl}furo[2,3-c]pyridin-2-yl)methanone dihydrochloride* (**PD4**).

Yeild: 1.15 g (66%). Light beige crystals, m.p. 243–247 °C (EtOH). ^1^H NMR (DMSO-*d*6): δ ppm 2.96 (s, 3H, CH_3_), 3.47–4.54 (m, 8H, H-2′, H-3′, H-5′, H-6′), 4.50 (s, 2H, CH_2_), 4.77 (s, 2H, CH_2_), 5.01 (br. s, 2H, 2NH^+^), 7.09 (s, 1H, H-3), 7.81 (d, *J* 8.3, 2H, H-2″, H-6″), 7.94 (s, 1H, H-5‴), 8.13 (d, *J* 8.6, 2H, H-3″, H-5″), 8.53 (s, 1H, H-6‴), 8.76–8.86 (m, 2H, H-5, H-2‴), 9.13 (s, 1H, H-4‴).). ^13^C NMR (75 MHz, DMSO-*_d6_*) δ 183.53 (C=O), 154.28 (C-O), 149.52, 149.14, 148.43, 144.52, 143.19, 135.46, 135.30, 132.46, 131.94 (2C), 131.70 (2C), 131.41, 126.45, 124.62, 123.68, 108.85, 61.71, 58.29, 52.21 (2C), 49.11 (2C), 18.49 (CH_3_). Anal.Calc for C_26_H_27_BrCl_2_N_4_O_2_;(%).: C, 54.00; H, 4.71; Br, 13.82; Cl, 12.26; N, 9.69%. Found: C, 54.06; H, 4.64; Br+Cl, 26.11; N, 9.57%.

*{4-[(1H-imidazol-1-yl)methyl]-7-methylfuro[2,3-c]pyridin-2-yl}(4-bromophenyl)methanone hydrochloride* (**PD5**).

Yield: 0.61 g (47%). Light beige crystals, m.p. 278–280 °C (EtOH). ^1^H NMR (DMSO-*d*6): δ ppm 2.87 (s, 3H, CH_3_), 5.89 (s, 2H, CH_2_), 7.10 (s, 1H, H-3), 7.60 (s, 1H, H-4′), 7.82 (d, *J* 8.4, 2H, H-3″, H-5″), 7.89 (s, 1H, NH^+^), 8.04 (d, *J* 8.5, 2H, H-2″, H-6″), 8.39 (s, 1H, H-5′), 8.72 (s, 1H, H-2′), 9.60 (s, 1H, H-5). ^13^C NMR (DMSO-*d*6) δ ppm: 16.267, 46.364, 113.426, 120.075, 120.736, 121.986, 124.275, 128.174, 131.565, 131.976, 134.457, 135.624, 138.606, 143.633, 149.820, 155.206, 182.194 (DMSO-*d*6: 38.831–40.082 Anal.Calc for C_19_H_15_BrClN_3_O_2_; (%).: C, 52.74; H, 3.49; Br, 18.47; Cl, 8.19; N, 9.71%. Found: C, 52.81; H, 3.29; Br+Cl, 26.80; N, 9.64%.

*(4-Bromophenyl)(4-{[4-(2-hydroxyethyl)piperazin-1-yl]methyl}-7-methylfuro[2,3-c]pyridin-2-yl)methanone* (**PD6**).

Yield: 1.00 g (73%). Light beige crystals, m.p. 105–107 °C (EtOH). ^1^H NMR (DMSO-*d*6): δ ppm 2.54 (s, 8H, H-2′, H-3′, H-5′, H-6′), 2.75 (s, 6H, CH_3_, CH_2_, OH), 3.54 (s, 2H, CH_2_), 3.79 (s, 2H, CH_2_), 7.77–7.82 (m, 2H, H-3″, H-5″), 7.87 (s, 1H, H-3), 7.98–8.03 (m, 2H, H-2″, H-6″), 8.23 (s, 1H, H-5). ^13^C NMR (DMSO-*d*6) δ ppm: 16.667, 47.502, 52.632, 115.417, 128.247, 131.716, 132.107, 134.697, 136.832, 141.641, 144.212, 149.804, 154.369, 161.108, 182.485 (DMSO-*d*6: 38.872–40.122). Anal.Calc for C_22_H_24_BrN_3_O_3_;(%).: C, 57.65; H, 5.28; Br, 17.43; N, 9.17%. Found: C, 57.58; H, 5.11; Br, 17.80; N, 9.04%.

*(4-Bromophenyl){4-[(4,5-dichloro-1H-imidazol-1-yl)methyl]-7-methylfuro[2,3-c]pyridin-2-yl}methanone* (**PD7**).

Yield: 1.30 g (93%). Light beige crystals, m.p. 183–185 °C (EtOAc: CH_3_CN 1:1). ^1^H NMR (DMSO-*d*6): δ ppm 2.77 (s, 3H, CH_3_), 5.56 (s, 2H, CH_2_), 7.78–7.82 (m, 2H, H-2″, H-6″), 7.91 (s, 1H, H-3), 7.98–8.01 (m, 2H, H-3″, H-5″), 7.60 (s, 1H, H-2′), 8.29 (s, 1H, H-5).). ^13^C NMR (DMSO-*d*6) δ ppm: 18.499, 44.750, 112.463, 113.385, 123.238, 124.937, 127.818, 131.117, 131.460, 131.917, 135.064, 136.543, 141.801, 144.369, 150.175, 152.896, 182.751 (DMSO-*d*6: 38.875–40.125). Anal.Calc. for C_19_H_12_BrCl_2_N_3_O_2_;(%).: C, 49.06; H, 2.60; Br, 17.18; Cl, 15.24; N, 9.03%. Found: C, 49.00; H, 2.41; Br+Cl, 32.51; N, 8.83%.

*(4-Bromophenyl){4-[(3,4-dihydroisoquinolin-2(1H)-yl)methyl]-7-methylfuro[2,3-c]pyridin-2-y})methanone* (**PD8**).

Yield: 1.04 g (75%). Light beige crystals, m.p. 134–135 °C (EtOAc). ^1^H NMR (DMSO-*d*6): δ ppm 2.77–2.85 (m, 7H, CH_3_, H-3′, H-4′), 3.65 (s, 2H, CH_2_), 3.97 (s, 1H, H-1′), 6.97 (s, 1H, H-3), 7.08–7.10 (m, 3H, H-5′–H-7′), 7.73 (d, *J* 8.1, 2H, H-2″, H-6″), 7.84–8.01 (m, 3H, H-8′, H-3″, H-5″), 8.31 (s, 1H, H-5).). ^13^C NMR (DMSO-*d*6) δ ppm: 18.424, 28.709, 50.176, 55.260, 56.652, 114.820, 125.471, 125.977, 126.387, 126.503, 127.663, 128.439, 131.395, 131.870, 132.248, 133.998, 134.614, 135.185, 142.052, 412.872, 150.381, 152.291, 182.851 (DMSO-*d*6: 38.878–40.128). Anal.Calc for C_25_H_21_BrN_2_O_2_; (%): C, 65.08; H, 4.59; Br, 17.32; N, 6.07%. Found: C, 65.01; H, 4.40; Br, 17.48; N, 6.03%.

*4-{[2-(4-Bromobenzoyl)-7-methylfuro[2,3-c]pyridin-4-yl]methyl}piperazine-1-carbaldehyde hydrochloride* (**PD9**).

Yield: 0.98 g (68%). Light beige crystals, m.p. 233–239 °C (EtOH). ^1^H NMR (DMSO-*d*6): δ ppm 2.91 (s, 3H, CH_3_), 3.51 (s, 8H, H-2′, H-3′, H-5′, H-6′), 4.74 (s, 2H, CH_2_), 7.81 (d, *J* 8.5, 2H, H-2″, H-6″), 8.10–8.14 (m, 2H, H-3″, H-5″), 8.45 (s, 1H, H-3), 8.75 (s, 1H, CH), 10.09 (s, 1H, H-5). ^13^C NMR (DMSO-*d*6) δ ppm: 18.309, 52.458, 53.228, 56.754, 58.447, 60.172, 114.820, 126.343, 127.612, 131.331, 131.838, 132.120, 135.191, 141.962, 142.661, 150.278, 152.189, 182.774 (DMSO-*d*6: 38.878–40.122). Anal.Calc. for C_21_H_21_BrClN_3_O_3_;(%).: C, 52.68; H, 4.42; Br, 16.69; Cl, 7.41; N, 8.78%. Found: C, 52.45; H, 4.29; Br+Cl, 24.21; N, 8.71%.

*3-{4-[(2-(4-bromobenzoyl)-7-methylfuro[2,3-c]pyridin-4-yl)methyl]piperazin-1-yl}propanenitrile* (**PD10**).

Yield: 1.18 g (84%). Light beige crystals, m.p. 129–132 °C (EtOH). ^1^H NMR (DMSO-*d*6): δ ppm 2.44–2.61 (m, 14H, CH_3_, H-2′, H-3′, H-5′, H-6′, DMF), 2.75 (s, 4H, 2CH_2_), 3.78 (s, 2H, CH_2_), 7.80 (d, *J* 8.3, 2H, H-2″, H-6″), 7.86 (s, 1H, H-3), 8.01 (d, *J* 8.2, 2H, H-3″, H-5″), 8.23 (s, 1H, H-5).^13^C NMR (DMSO-*d*6) δ ppm: 14.877, 18.288, 52.117–52.220(d), 52.585, 56.554, 114.768, 119.833, 126.200, 127.585, 131.310, 131.817, 132.150, 135.164, 142.005, 142.672, 150.245, 152.175, 182.766 (DMSO-*d*6: 38.832–40.082). Anal.Calc. for C_23_H_23_BrN_4_O_2_;(%).: C, 59.11; H, 4.96; Br, 17.10; N, 11.99%. Found: C, 59.03; H, 4.89; Br, 17.29; N, 11.72%.

*(4-Bromophenyl)(7-methyl-4-{[(pyridin-3-ylmethyl)amino]methyl}furo[2,3-c]pyridin-2-yl)methanone dihydrochloride* (**PD11**).

Yield: 0.67 g (44%). Light beige crystals, m.p. 245–248 °C (EtOH). ^1^H NMR (DMSO-*d*6): δ ppm 2.93 (s, 3H, CH_3_), 4.48 (s, 2H, CH_2_), 4.66 (s, 2H, CH_2_), 5.69 (br. s, 2H, 2NH^+^), 7.77–7.83 (m, 2H, H-2″, H-6″), 7.88 (s, 1H, H-3), 8.12–8.17 (m, 2H, H-3″, H-5″), 8.48 (s, 1H, H-5′), 8.74–8.82 (m, 3H, H-2′, H-4′, H-6′), 9.11 (s, 1H, H-5). ^13^C NMR (75 MHz, DMSO-*_d6_*) δ 164.38 (C=O), 163.41 (2C), 143.04 (2C), 141.77 (2C), 141.14, 140.42, 139.64, 129.99 (3C), 126.22 (4C), 115.70, 111.85, 57.36 (CH_2_-NH), 56.48 (NH-CH_2_), 18.98 (CH_3_). Anal.Calc. for C_22_H_20_BrCl_2_N_3_O_2_; (%).: C, 51.89; H, 3.96; Br, 15.69; Cl, 13.92; N, 8.25%. Found: C, 51.73; H, 3.71; Br+Cl, 29.81; N, 8.04%.

*(2,3-Dihydrobenzo[d][1,4]dioxin-6-yl)(4-{[(3,4-dimethoxybenzyl)amino]methyl}-7-methylfuro[2,3-c]pyridin-2-yl)methanone hydrochloride* (**PD12**).

Yield: 0.94 g (61%). Light beige crystals, m.p. 208–211 °C (EtOH). ^1^H NMR (DMSO-*d*6): δ ppm 2.97 (s, 3H, CH_3_), 3.77 (s, 3H, OCH_3_), 3.80 (s, 3H, OCH_3_), 4.16 (s, 2H, CH_2_), 4.33–4.42 (m, 4H, H-2″, H-3″), 4.57 (s, 2H, CH_2_), 5.69 (br. s, 1H, NH^+^), 6.86 (d, *J* 8.2, 1H, H-8″), 7.03–7.06 (m, 2H, H-5′, H-6′), 7.63 (d, *J* 2.1, 1H, H-2′), 7.78 (dd, 8.5, *J* 2.2, 1H, H-7″), 8.32 (s, 1H, H-3), 8.82 (s, 1H, H-5″), 10.41 (s, 1H, H-5). ^13^C NMR (75 MHz, DMSO-*_d6_*) δ 186.65 (C=O), 169.44 (C-C=O), 164.38, 162.00 (C-CH_3_), 157.81, 142.07 (2C), 140.19 (2C), 127.65 (4C), 122.99 (4C), 105.07, 96.84, 95.57, 57.90 (CH_2_-O-O-CH_2_), 56.89 (CH_2_-O-O-CH_2_), 56.47 (4C), 18.99 (CH_3_). Anal.Calc. for C_27_H_27_ClN_2_O_6_;(%).: C, 63.47; H, 5.33; Cl, 6.94; N, 5.48%. Found: C, 63.30; H, 5.16; Cl, 6.99; N, 5.33%.

*(2,3-Dihydrobenzo[d][1,4]dioxin-6-yl){7-methyl-4-[(4-(pyridin-3-ylmethyl)piperazin-1-yl)methyl]furo[2,3-c]pyridin-2-yl}methanone dihydrochloride* (**PD13**).

Yield: 0.97 g (58%). Light beige crystals, m.p. 224–227 °C (EtOH). ^1^H NMR (DMSO-*d*6): δ ppm 2.96 (s, 3H, CH_3_), 3.39–3.46 (m, 8H, H-2′, H-3′, H-5′, H-6′), 4.33–4.43 (m, 6H, H-2″, H-3″, CH_2_), 4.70 (m, 2H, CH_2_), 5.48 (br. s, 2H, 2NH^+^), 7.00–7.10 (m, 2H, H-3, H-8″), 7.62 (d, *J* 2.1, 1H, H-5″), 7.76 (dd, 8.5, *J* 2.1, 1H, H-7″), 8.39 (s, 1H, H-5‴), 8.65 (s, 1H, H-4‴), 8.77 (s, 1H, H-2‴), 8.82 (d, *J* 5.2, H-6‴), 9.06 (s, 1H, H-5). ^13^C NMR (DMSO-*d*6) δ ppm: 15.728, 47.653, 63.875, 64.645, 113.920, 117.511, 118.498, 124.179, 126.519, 128.545, 137.823, 143.107, 143.376, 144.678, 146.819, 148.987, 149.487, 155.700, 180.995 (DMSO-*d*6: 38.741–39.997). Anal.Calc. for C_28_H_30_Cl_2_N_4_O_4_; (%).: C, 60.33; H, 5.42; Cl, 12.72; N, 10.05%. Found: C, 60.16; H, 5.27; Cl, 12.87; N, 9.83%.

*(2,3-Dihydrobenzo[d][1,4]dioxin-6-yl)(4-{[4-(2-hydroxyethyl)piperazin-1-yl]methyl}-7-methylfuro[2,3-c]pyridin-2-yl)methanone dihydrochloride* (**PD14**).

Yield: 0.97 g (75%). Light beige crystals, m.p. 252–255 °C (EtOH). ^1^H NMR (DMSO-*d*6): δ ppm 3.00 (s, 3H, CH_3_), 3.27 (t, *J* 4.9, 2H, CH_2_), 3.62–3.71 (m, 8H, H-2′, H-3′, H-5′, H-6′), 3.85 (t. *J* 5.0, 2H, CH_2_), 4.34–4.42 (m, 4H, H-3″, H-8″), 4.82 (s, 2H, CH_2_), 5.71 (br. s, 3H, OH, 2NH^+^), 7.04 (d, *J* 8.5, 1H, H-8″), 7.62 (d, *J* 2.1, 1H, H-5″), 7.78 (dd, *J* 8.5, 2.1, 1H, H-7″), 8.46 (s, 1H, H-3), 8.85 (1H, H-5. ^13^C NMR (400 MHz, DMSO-*d*_6_) δ 15.89, 55.09, 63.87, 64.64, 113.98, 117.53, 118.49, 124.16, 128.60, 137.53, 143.26, 143.38, 148.97, 149.50, 155.52, 181.05. Anal.Calc. for C_24_H_29_Cl_2_N_3_O_5_;(%).: C, 56.48; H, 5.73; Cl, 13.89; N, 8.23%. Found: C, 56.31; H, 5.61; Cl, 13.97; N, 8.10%.

### 3.3. Computational Studies

#### 3.3.1. System Preparation

The three-dimensional structure of wild-type (PDB ID: 1M17) [52], L858R/T790M (PDB ID: 4I22) [53] and L858R/T790M/C797S EGFR (PDB ID: 6LUD) [52] were obtained from the Protein Data Bank. These crystal structures complexed with known drugs and subsequently known drugs were extracted. A series of 14 furo[2,3-c]pyridine derivatives was built using the Gaussview 09 program. All the ligands were optimized using the Gaussian 09 program (HF/6-31G*) and, subsequently, the protonation states of all ionizable amino acids were characterized using ChemAxon at pH 7.0. Molecular docking studies were performed using the GOLD program. The setting of the docking was studied using the following parameter as 10 Å with a sphere and 100 docking poses. The docking runs in each complex were sorted and selected based on GOLD fitness score. Then, the docking results were visualized for interaction using the UCSF Chimera package. The docked PDs/EGFRs with the highest GOLD fitness scores were chosen as the initial structures for performing MD simulations.

#### 3.3.2. Molecular Dynamics Simulation and Free Energy Calculation Based on Solvated Interaction Energy (SIE) Method

The initial structures of PDs/EGFRs derived from molecular docking were carried out using pmemd CUDA in the AMBER 16 program [54] under periodic boundary conditions with the isobaric isothermal (NPT) ensemble in triplicate. The general AMBER force fields GAFF [55] and FF14SB [56] were applied for the ligand and protein, respectively. The partial charges of ligand were generated as follows: (i) the 3D structure of **PD** derivatives were optimized using the HF/6-31G* method [57] in the Gaussian09 program; (ii) the electrostatic potential (ESP) charge and restrained ESP (RESP) charge of the ligand were obtained by the AMBER16 program. All studied complexes were solvated by the TIP3P water model [58]. All missing hydrogen atoms of the protein and ligand were added by the LEaP module and subsequently minimized. The sodium ions were randomly neutralized in the simulated systems by Cl^−^ counter ions. The added hydrogen atoms and water molecules were then minimized using 2500 steps of the steepest descents (SD), followed by 2500 steps of the conjugated gradient (CG), before starting the MD simulations. The SHAKE algorithm was used to fix all covalent bonds involving hydrogen atoms [59]. The systems were heated up to 310 K for 100 ps. Finally, the whole system was performed under the NPT ensemble (310 K, 1 atm) until reaching 100 ns. The root mean square deviation (RMSD) of each system was calculated using all-atom, and then the MD trajectories from the last 10-ns simulation were extracted for analysis in terms of total binding free energy (ΔG_bind_) based on the solvated interaction energy (SIE) method. The potent **PD** derivatives with the lowest binding free energy were selected to determine EGFR inhibitory as well as anticancer activity. After finding the more potent **PD** derivative obtained from in silico screening and in vitro evaluation, the 500-ns MD simulation was performed. In addition, the per-residue decomposition free energy (ΔG_bind,residue_) calculations were carried out using the MMPBSA.py module in AMBER16 [60]. The CPPTRJ module was used to calculate intermolecular hydrogen bonding.

### 3.4. Biological Studies

#### 3.4.1. EGFR Inhibitory ACTIVITY

The IC_50_ values of the potent PD derivatives obtained from the SIE method were measured using the ADP-Glo^TM^ kinase assay kit as previously reported [19]. Firstly, 8 µL of buffer (40 mM Tris-HCl pH 7.5, 20 mM MgCl_2_, and 0.1 mg/mL bovine serum albumin) were added to a 384-well plate (Promega, solid white). Secondly, 5 µL of 1.25 ng/µL EGFRs enzyme (wild-type, L858R/T790M, and L858R/T790M/C797S EGFR) and 2 µL of 1 μM of compounds/known drugs were added separately to individual cell cultures, followed by 10 µL of a mixture of 5 µM ATP and 2.5 µM poly(glu-tyr), and were incubated for 1 h at room temperature. Thirdly, 5 µL of the ADP-Glo reagent was added and incubated for 40 min. Finally, 10 µL of kinase detection reagent was added and incubated at room temperature for 30 min and was detected by measuring the luminescence using a microplate reader (Infinite M200 microplate reader, Tecan, Männedorf, Switzerland). A triplicate assay was performed on all potent **PD** derivatives and known drugs. The percentage relative inhibition (%) of potent compounds was measured compared to the control with no inhibitor as shown in Equation (1):

(1) $$\%\mathrm{Relative}\;\mathrm{inhibition}=\frac{\left(\mathrm{positive}-\mathrm{negative}\right)-\left(\mathrm{sample}-\mathrm{negative}\right)}{\left(\mathrm{positive}-\mathrm{negative}\right)}\times 100.$$

#### 3.4.2. Cell Viability Assay

The cytotoxicity activities of potent **PD** derivatives obtained from the SIE method against A549 and H1975, as well as Vero (which are adherent cells), were assessed using MTT (3-(4,5-dimethyl-2-thiazolyl)-2,5-diphenyl-2H-tetrazolium bromide) assays. The A549 and Vero cells were grown in complete DMEM medium whereas H1975 cells were grown in complete RPMI medium supplemented with 10% (*v*/*v*) FBS, 100 U/mL penicillin and 100 µg/mL streptomycin. All cells were routinely cultured at 37 °C in a 5% (*v*/*v*) CO_2_, 95% (*v*/*v*) air humidified incubator. A549 (5 × 10^3^ cells per well), H1975 (5 × 10^3^ cells per well), and Vero (2 × 10^3^ cells per well) cells were seeded into 96-well plates and incubated overnight. After cell attachment, cells were replenished using fresh medium with 0.1% FBS containing various concentrations of potent PD derivatives and known drugs and then incubated at 37 °C for 72 h. After that, fresh medium containing MTT solution (5 mg/mL) was added and incubated at 37 °C. After 3 h, the formed formazan crystal was dissolved with 100 μL of dimethyl sulfoxide (DMSO). Finally, the absorbance of formazan solution was measured at a wavelength 570 nm using a microplate reader (Infinite M200 microplate reader, Tecan, Männedorf, Switzerland). The independent treatments were repeated in triplicate. The IC_50_ determination of compounds was achieved using GraphPad Prism version 9.0.

## 4. Conclusions

In this work, a combination of in silico and in vitro studies were performed to screen for novel EGFR inhibitors that could inhibit both wild-type and mutant forms of EGFR. We found that potent compounds from virtual screening exhibited promising EGFR inhibitory activity. In particular, **PD13** showed strong inhibition of wild-type and L858R/T790M double mutant EGFR, while L858R/T790M/C797S showed moderate inhibition. In addition, the most potent **PD13** revealed high cytotoxicity against A549 and H1975. In 500-ns MD simulations, the model of **PD13** and known drugs/EGFRs was stable. The binding affinity of **PD13** complexed with wild-type and mutant forms of EGFR was significantly higher than that of known drugs (except for the L858R/T790M/C797S system), which was consistent with the hot-spot residues and EGFR inhibitory activity. The $\Delta G_{\mathrm{bind}}^{\mathrm{residue}}$ calculations revealed that M793 and S797 are the main residues in the hinge region for the binding of **PD13** with three EGFR strains via H-bond formations. In addition, the binding of all studied **PD** compounds is driven mainly by vdW interaction. Our results showed that **PD13** could potentially act as a promising non-mutant and mutant EGFR drug for cancer therapy.

## Figures and Tables

**Table 1:** Chemical structure of furopyridine; **PD** and its derivatives.

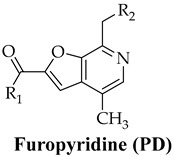					
Cipher	R_1_	R_2_	Cipher	R_1_	R_2_
**PD1**	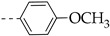	OH	**PD9**	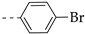	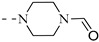
**PD2**	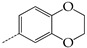	OH	**PD10**	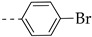	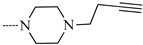
**PD3**	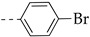	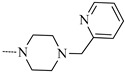	**PD11**	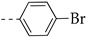	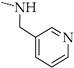
**PD4**	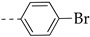	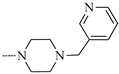	**PD12**	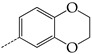	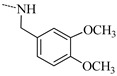
**PD5**	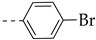	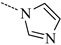	**PD13**	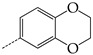	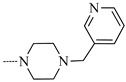
**PD6**	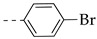	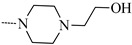	**PD14**	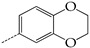	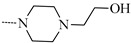
**PD8**	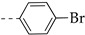	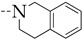			

**Table 2:** EGFR inhibitory activities of focused **PD** and drugs in different forms of EGFR.

Compounds	IC_50_ (nM)		
	Wild-Type	L858R/T790M	L858R/T790M/C797S
**PD4**	4.78 ± 0.73	91.02 ± 2.01	103.70 ± 3.62
**PD13**	11.64 ± 1.30	10.51 ± 0.71	21.93 ± 1.79
Erlotinib	14.11 ± 0.19	87.96 ± 0.89	108.50 ± 1.54
Afatinib	78.19 ± 1.20	20.14 ± 0.58	23.86 ± 2.02
Osimertinib	441.90 ± 3.02	17.39 ± 0.25	8.98 ± 0.53

**Table 3:** In vitro anticancer activity of focused PD and drugs against NSCLC and normal cell lines.

Compounds	IC_50_ (μM)		
	A549	H1975	Vero
**PD4**	19.70 ± 0.59	27.26 ± 1.02	>50
**PD13**	18.09 ± 1.57	33.87 ± 0.86	>100
Erlotinib	19.43 ± 0.79	41.84 ± 2.19	>30
Afatinib	34.69 ± 0.67	27.33 ± 1.87	>50
Osimertinib	25.28 ± 1.21	18.33 ± 2.00	>50

**Table 4:** The average ΔG_bind_ and its energy component (kcal/mol) of **PD13** and drugs in complexes with EGFRs calculated with the MM/PBSA method, compared to the half-maximal inhibitory concentration (IC_50_) values. Data are shown as mean ± standard deviation (SD) of three independent simulations.

	ΔE_ele_	ΔE_vdW_	ΔE_MM_	TΔS	ΔG_sol (PBSA)_	ΔG_bind (PBSA)_	IC_50_ (nM)	ΔG_bind,exp_
**Wild-type**								
**Erlotinib**	−15.43 ± 1.02	−65.54 ± 1.52	−80.98 ± 1.87	−22.69 ± 0.94	45.70 ± 0.96	−12.57 ± 0.69	11.44	10.83
**PD13**	−25.46 ± 2.01	−98.54 ± 0.89	−124.01 ± 0.71	−27.98 ± 0.75	77.69 ± 0.05	−18.33 ± 0.42	14.11	10.70
**L858R/T790M/58**								
**Afatinib**	−15.56 ± 0.85	−60.87 ± 0.66	−79.44 ± 0.09	−20.47 ± 0.99	39.23 ± 0.12	−19.73 ± 0.95	20.14	10.49
**PD13**	−14.36 ± 0.83	−91.64 ± 1.99	−106.00 ± 3.54	−10.21 ± 0.87	73.11 ± 0.93	−22.68 ± 0.47	10.51	10.88
**L858R/T790M/C797S**								
**Osimertinib**	−20.03 ± 0.79	−61.65 ± 1.20	−81.68 ± 0.28	−19.24 ± 0.88	42.57 ± 0.45	−19.87 ± 0.84	8.98	10.97
**PD13**	−12.02 ± 1.44	−94.02 ± 0.98	−106.04 ± 0.95	−18.21 ± 1.55	71.11 ± 0.69	−16.71 ± 0.69	21.93	10.44

**Table 5:** Reaction conditions and yields of compounds **PD3–PD14**.

Furopyridine	Solvent (mL)	Reagent R_3_NH (mmol)	Reaction Temperature (°C)	Reaction Time (h)	Yield (%)
**PD3**	EtOH 10	3	25–30	6.0	62
**PD4**	EtOH 10	3	25–30	6.0	66
**PD5**	EtOH 10	12	boiling	3.0	47
**PD6**	EtOH 10	10	25–30	6.0	73
**PD7 ^1^**	DMF 8	3	35–40	3.0	93
**PD8**	DMF 10	3	45–50	6.0	75
**PD9**	EtOH 8, H_2_O 2	3	boiling	3.0	68
**PD10**	EtOH 10	3	40–45	5.0	84
**PD11**	EtOH 10	3	35–40	5.0	44
**PD12**	DMF 5	3	30–40	5.0	61
**PD13 ^2^**	DMF 5	3.5	30–40	5.0	58
**PD14 ^2^**	DMF 5	3.5	30–40	5.0	75

Note: 1. For compound synthesis, **PD7** NaH (3.5 mmol) was used instead of K_2_CO_3_. 2. For compounds synthesis, **PD13, PD14** NaHCO_3_ (6 mmol) was used instead of K_2_CO_3_.

## Data Availability

Not applicable.

## Supplementary Materials

The following supporting information can be downloaded at: https://www.mdpi.com/article/10.3390/molecules28073014/s1, ^1^H NMR Spectra of compounds **PD1**–**PD14**.

## Sample Availability

Samples of the compounds are available from the authors.
