# Supplementary material for: Discovery of Novel EGFR Inhibitor Targeting Wild-Type and Mutant Forms of EGFR: In Silico and In Vitro Study
Source: Molecules. 2023 Mar 28;28(7):3014. doi: 10.3390/molecules28073014 (PMC10096398; doi:10.3390/molecules28073014)

Electronic supplementary materials *Molecules*, 2023

Discovery of novel EGFR inhibitor targeting wild-type and mutant forms of EGFR: *in silico* and *in vitro* study

Duangjai Todsaporn, Alexander Zubenko, Victor Kartsev, Thitinan Aiebchun, Panupong Mahalapbutr, Anthi Petrou, Athina Geronikaki, Liudmila Divaeva, Victoria Chekrisheva, Ilkay Yildiz, Kiattawee Choowongkomon, Thanyada Rungrotmongkol

Table of Contents

General Information

^{1}H NMR Spectra of compounds **PD1-PD14**

^{13}C NMR Spectra of compounds **PD1-PD14**

General Information

NMR ^{1}H of new compounds were recorded on a spectrometer Bruker AC 300 (300 MHz) in DMSO-d_6. Chemical shifts of nuclei ^{1}H and ^{13}C were measured relatively the residual signals of deuterated solvent (δ =2.50 ppm for protons and 39.5 ppm for carbon nuclei). Coupling constants (*J*) are reported in Hz.

^{1}H NMR Spectra of compounds PD1-PD14.

[4-(Hydroxymethyl)-7-methylfuro[2,3-c]pyridin-2-yl](4-methoxyphenyl)methanone (**PD1**).

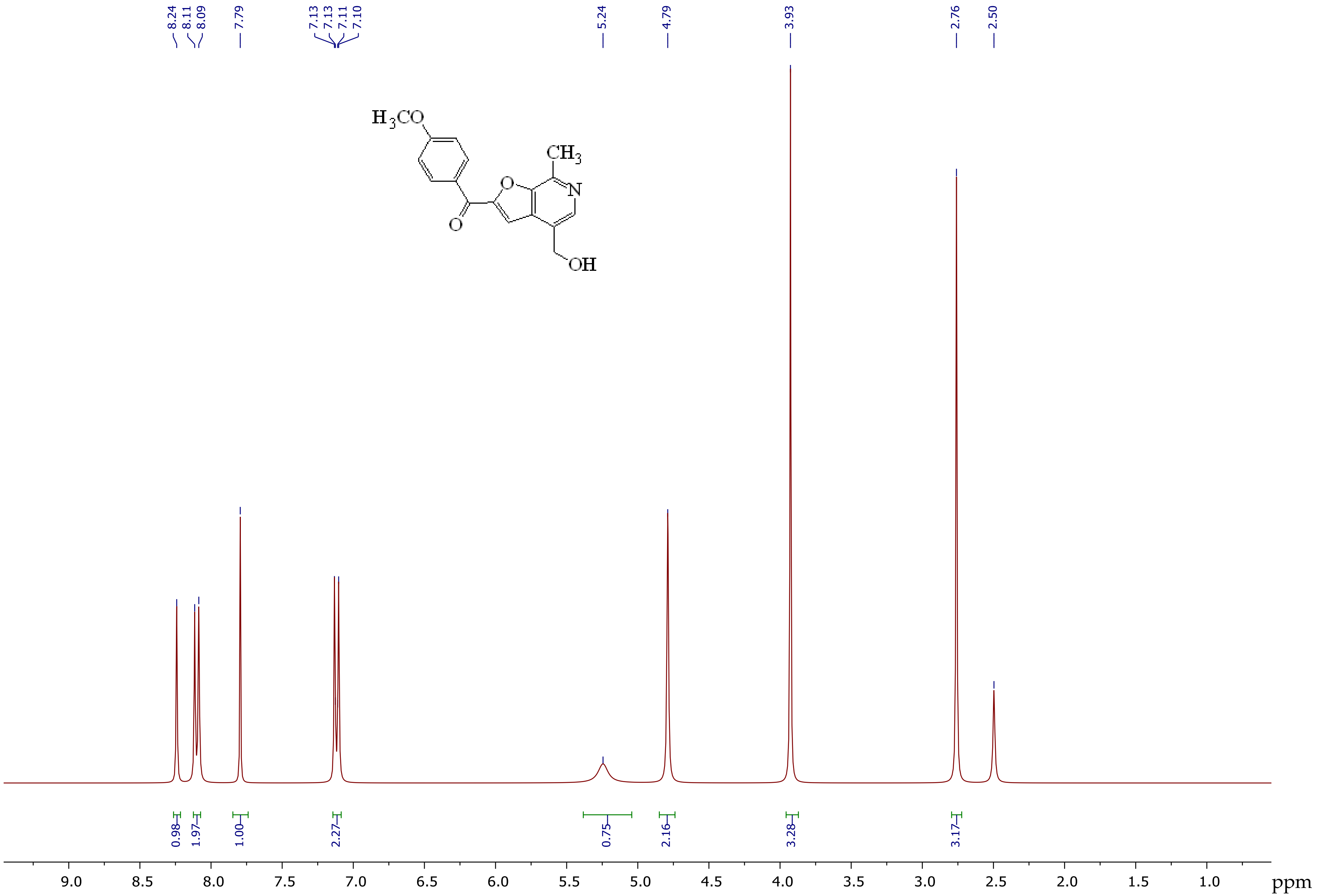

(1,4-Dihydrobenzo[d][1,2]dioxin-6-yl)(4-(hydroxymethyl)-7-methylfuro[2,3-c]pyridin-2-yl)methanone (**PD2**)

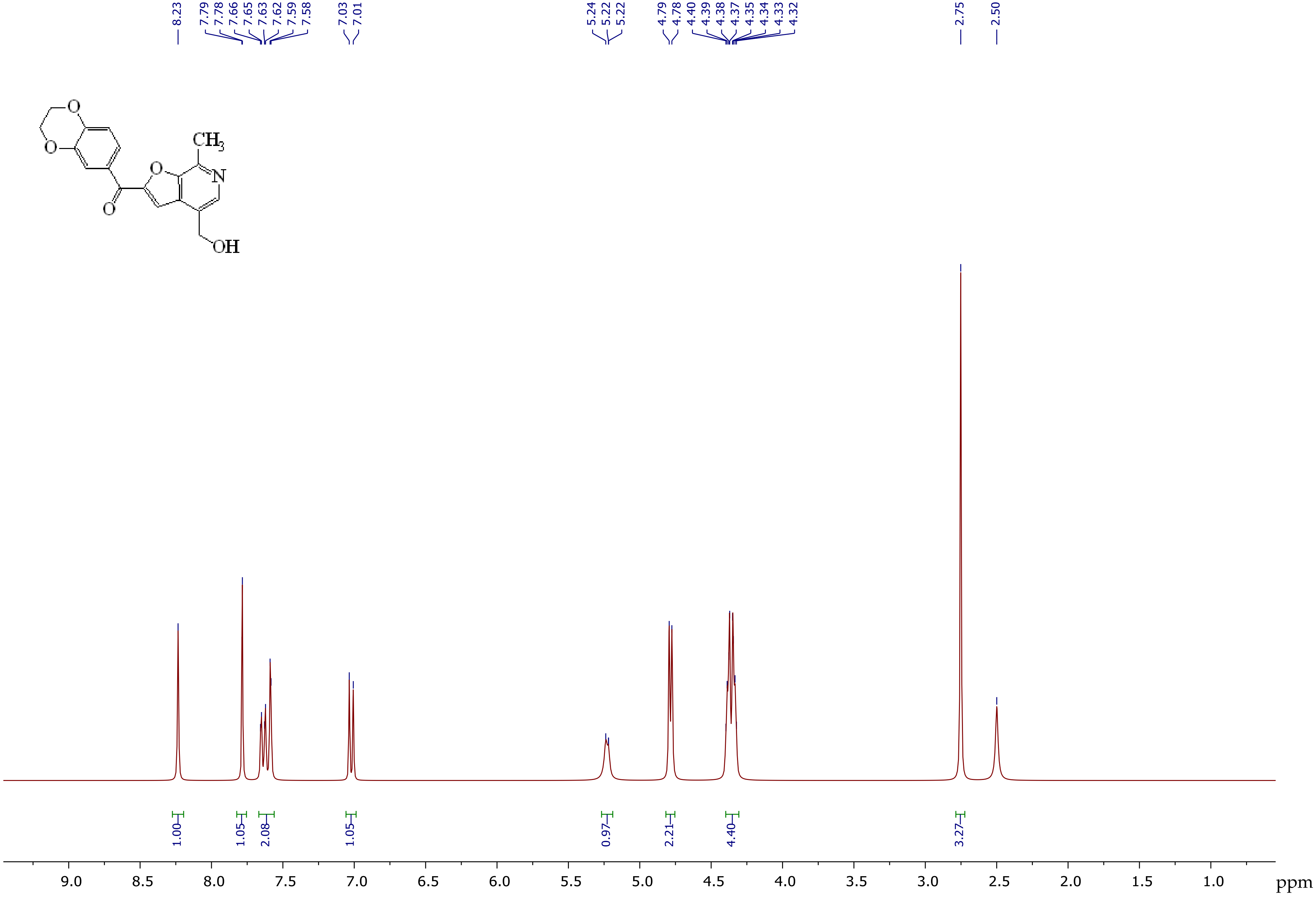

(4-Bromophenyl){7-methyl-4-[(4-(pyridin-2-ylmethyl)piperazin-1-yl]methyl}furo[2,3-c]pyridin-2-yl)methanone dihydrochloride (**PD3**).

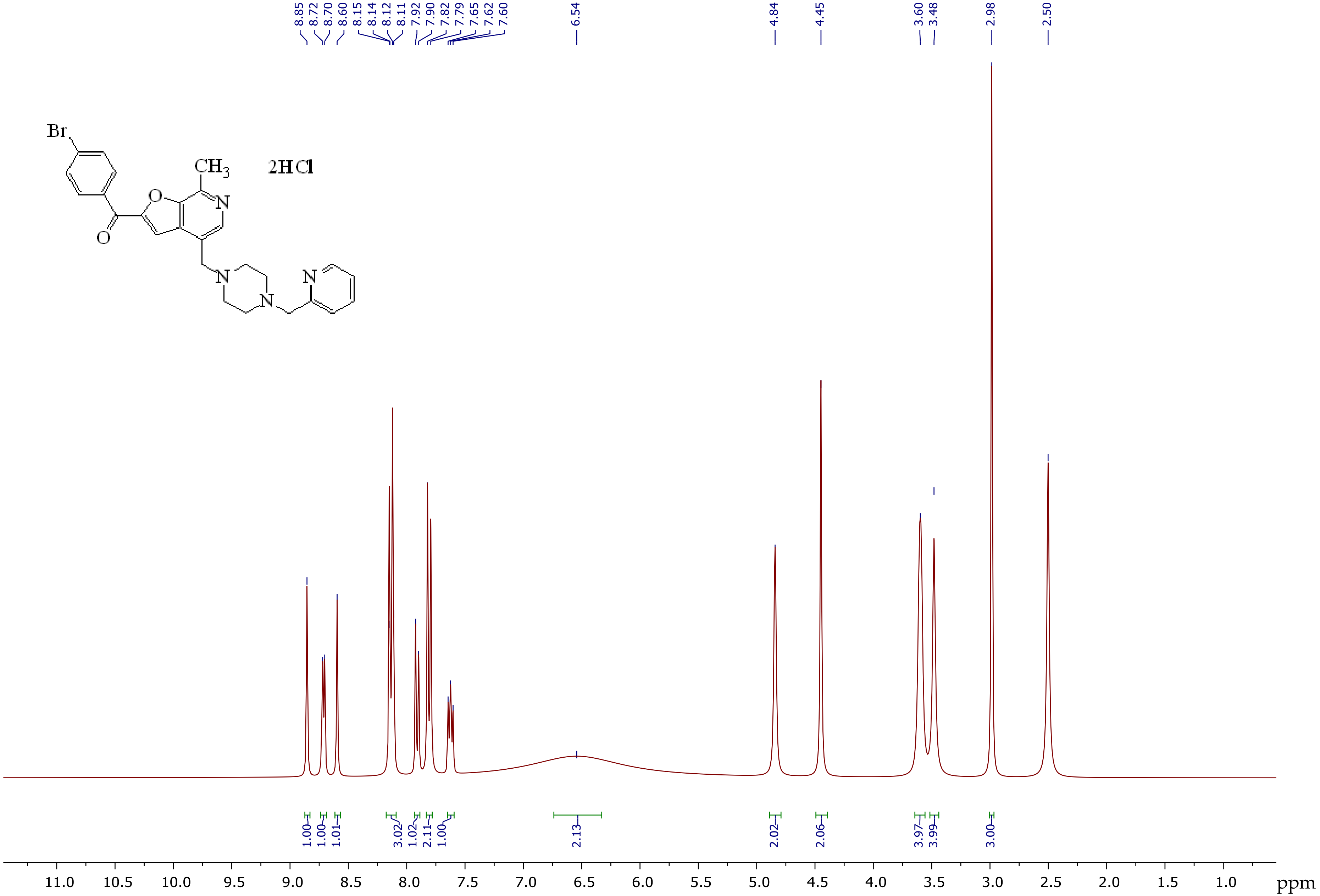

(4-Bromophenyl)(7-methyl-4-((4-(pyridin-3-ylmethyl)piperazin-1-yl)methyl)furo[2,3-c]pyridin-2-yl)methanone (**PD4**).

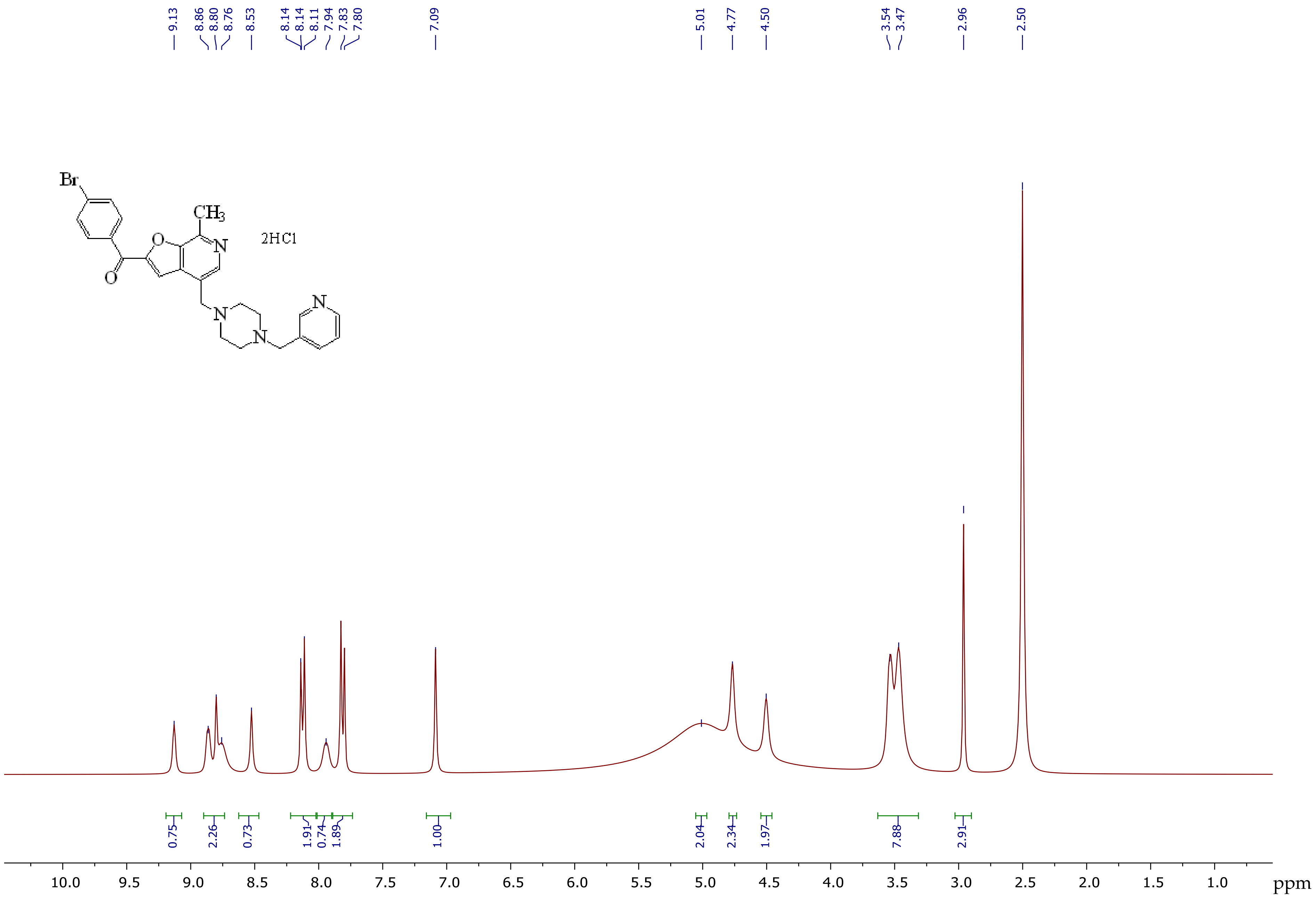

(4-((1H-Imidazol-1-yl)methyl)-7-methylfuro[2,3-c]pyridin-2-yl)(4-bromophenyl)methanone hydrochloride (**PD5**).

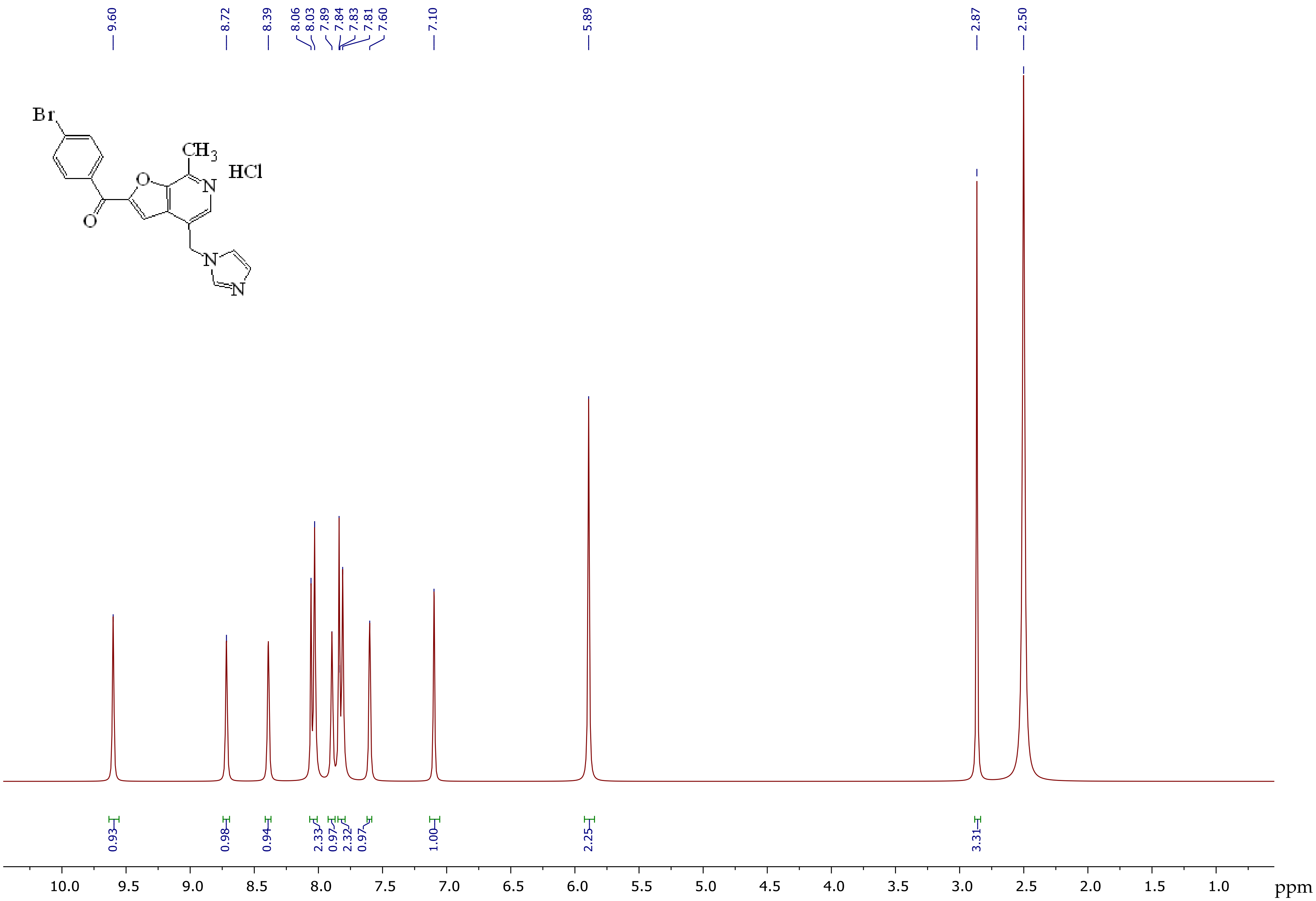

(4-Bromophenyl)(4-((4-(2-hydroxyethyl)piperazin-1-yl)methyl)-7-methylfuro[2,3-c]pyridin-2-yl)methanone (**PD6**).

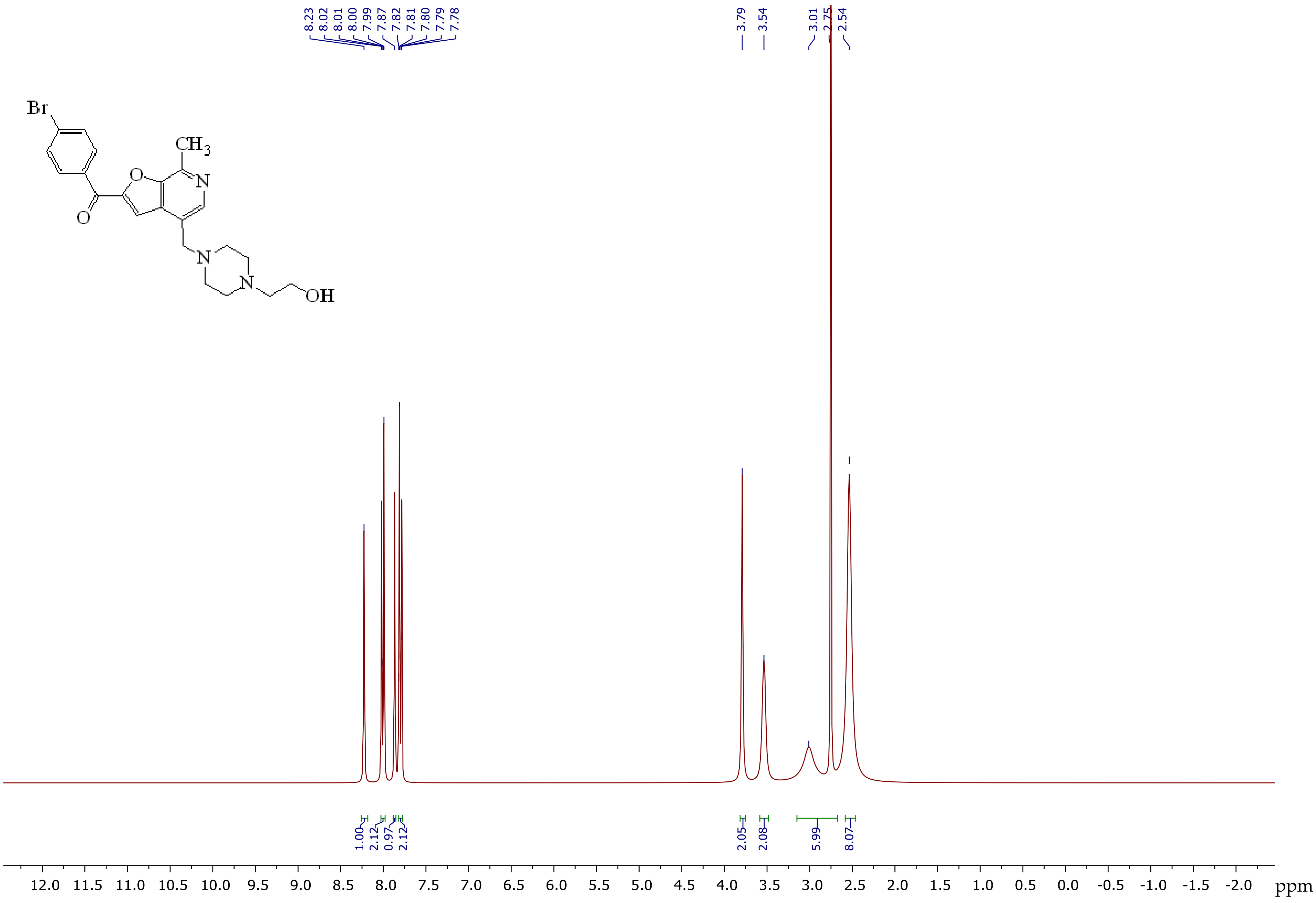

(4-Bromophenyl)(4-((4,5-dichloro-1H-imidazol-1-yl)methyl)-7-methylfuro[2,3-c]pyridin-2-yl)methanone (**PD7**).

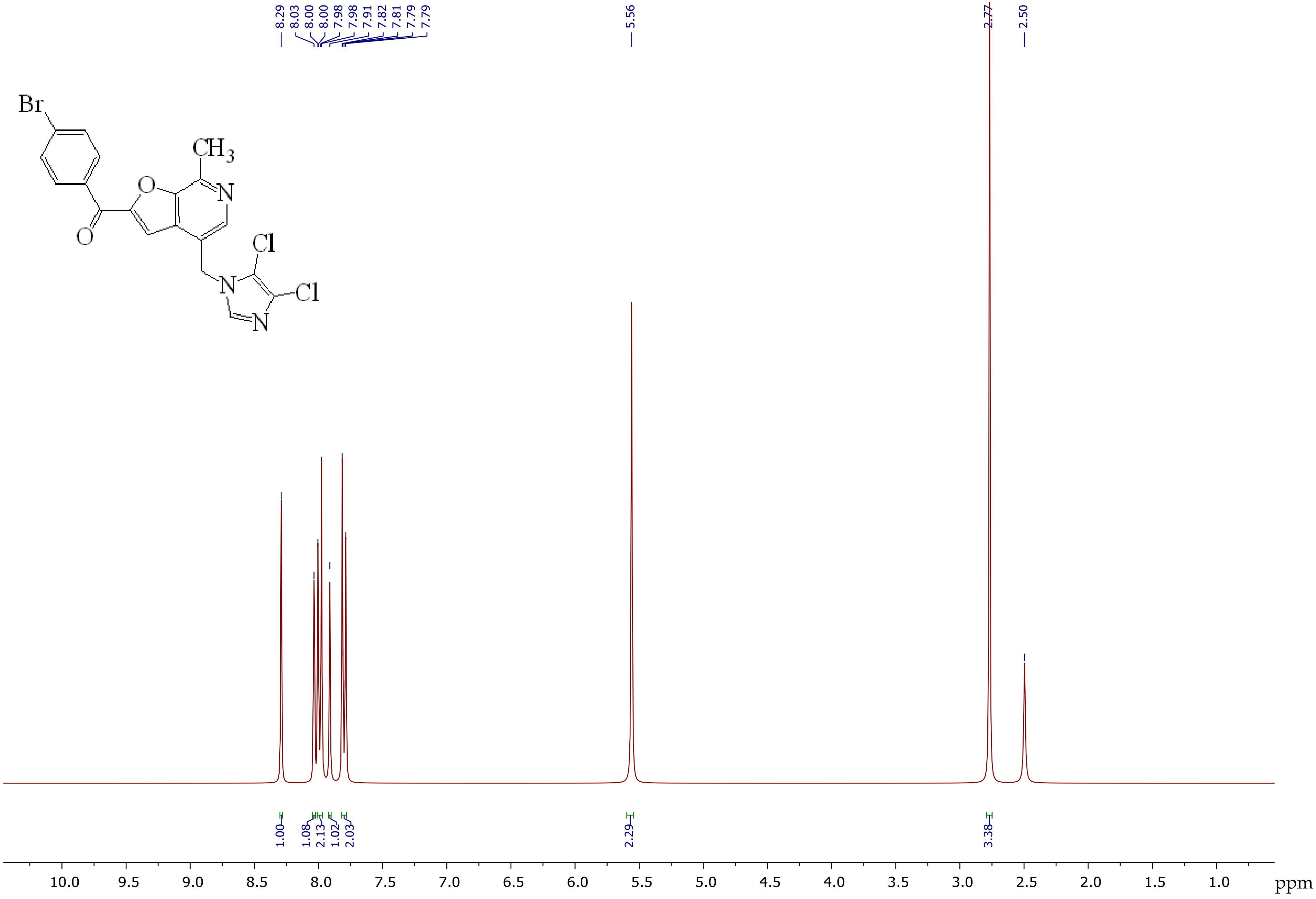

(4-Bromophenyl)(4-((3,4-dihydroisoquinolin-2(1H)-yl)methyl)-7-methylfuro[2,3-c]pyridin-2-yl)methanone (**PD8**).

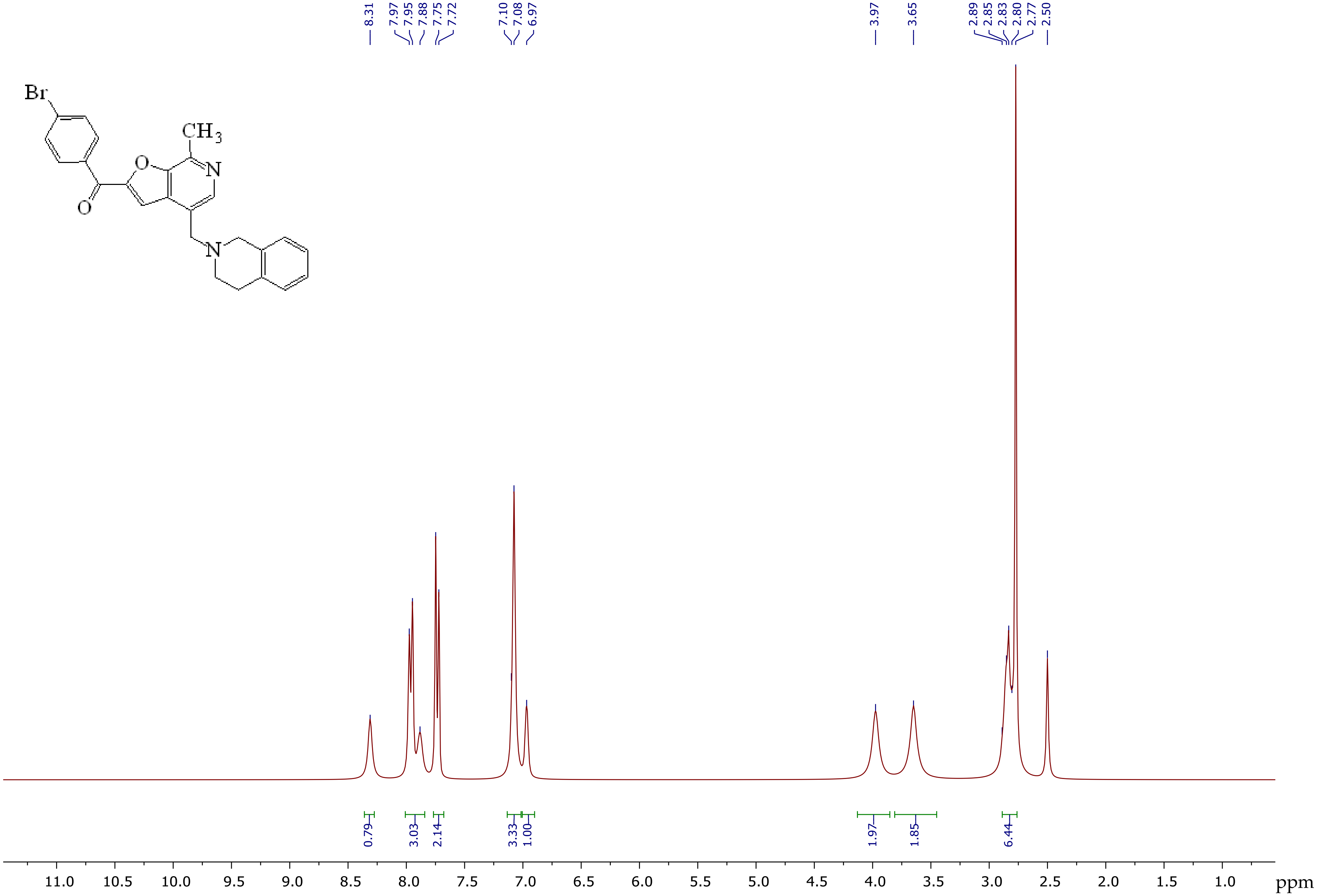

4-((2-(4-Bromobenzoyl)-7-methylfuro[2,3-c]pyridin-4-yl)methyl)piperazine-1-carbaldehyde hydrochloride (**PD9**).

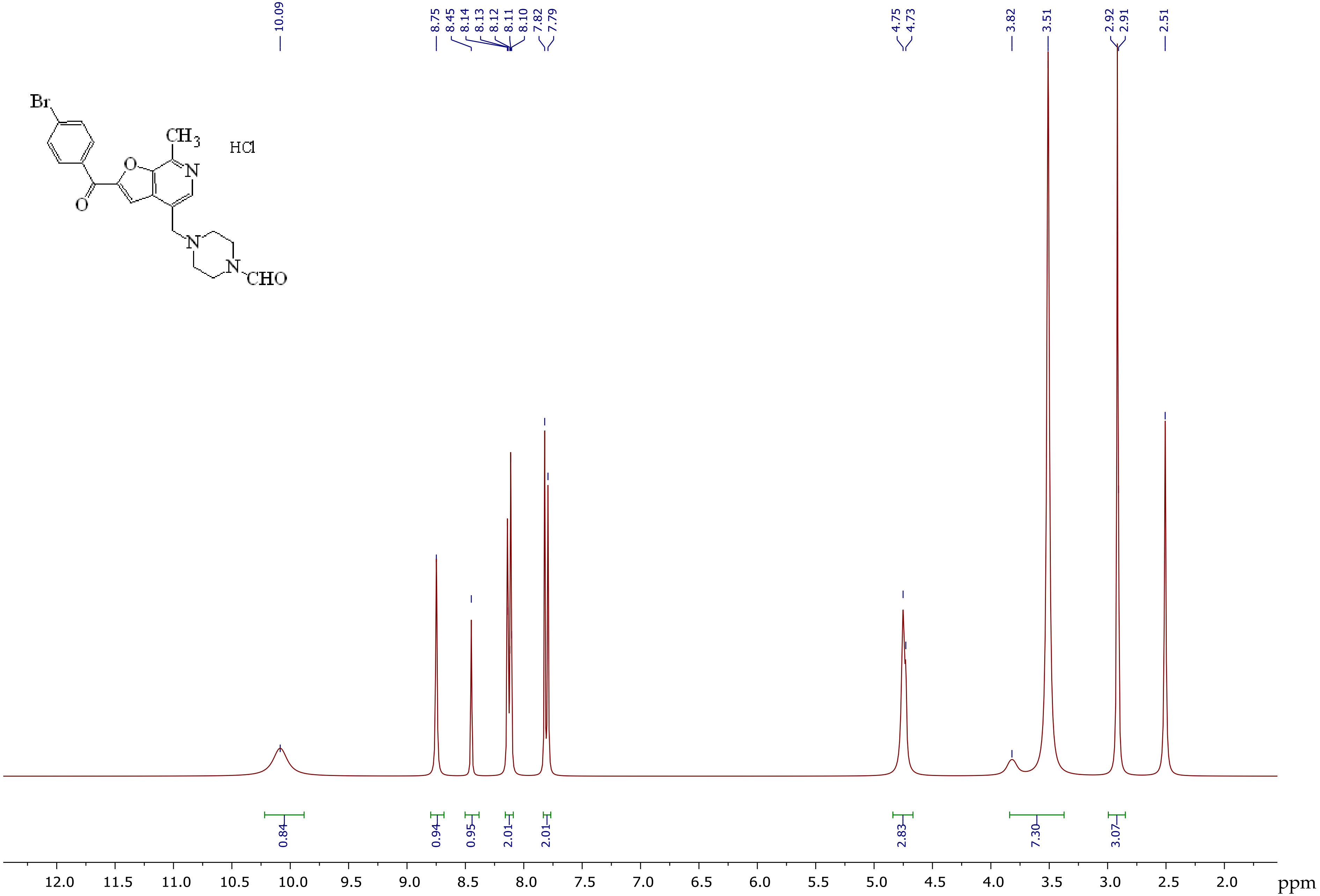

3-(4-((2-(4-Bromobenzoyl)-7-methylfuro[2,3-c]pyridin-4-yl)methyl)piperazin-1-yl)propanenitrile (**PD10**).

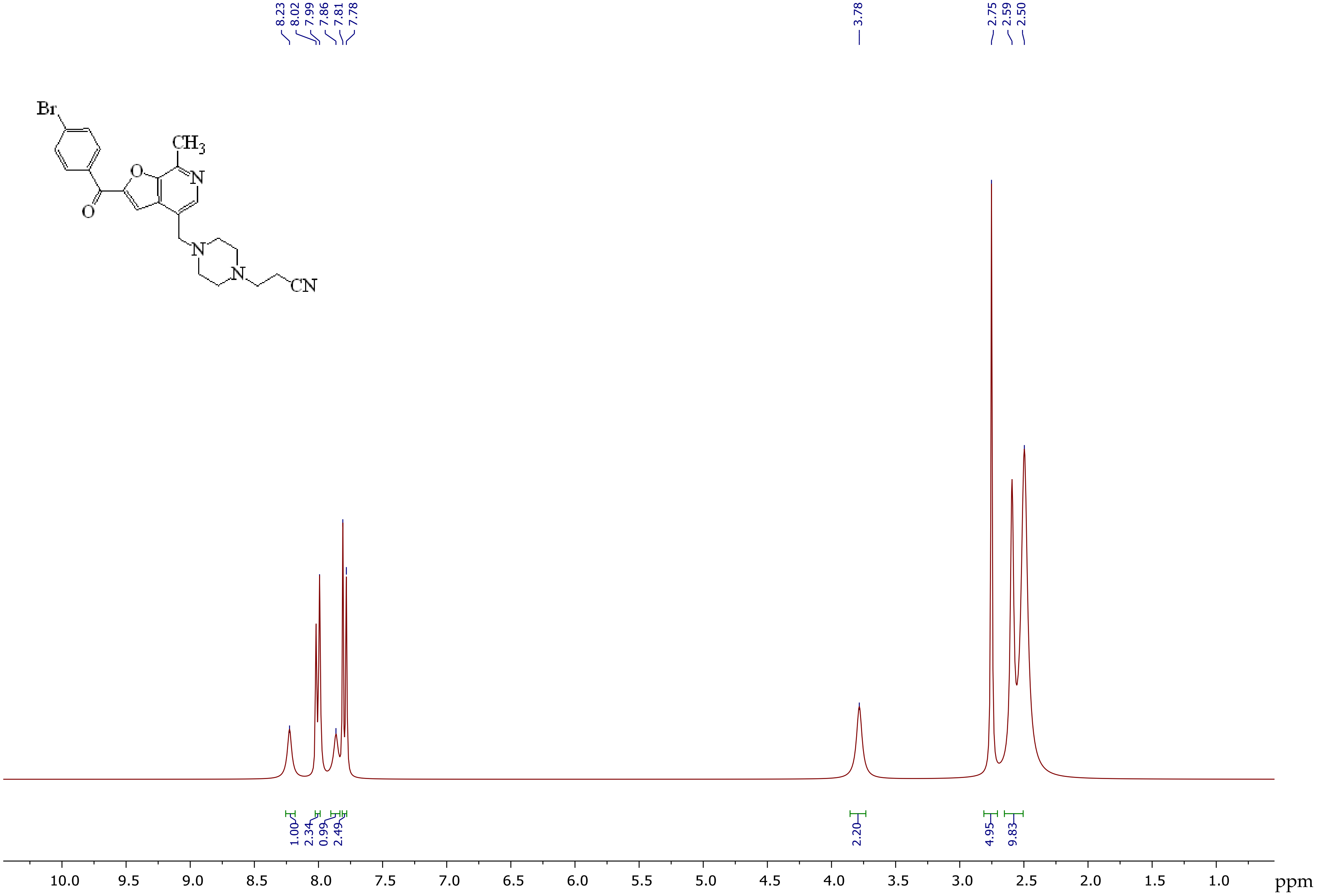

(4-Bromophenyl)(7-methyl-4-(((pyridin-3-ylmethyl)amino)methyl)furo[2,3-c]pyridin-2-yl)methanone dihydrochloride (**PD11**).

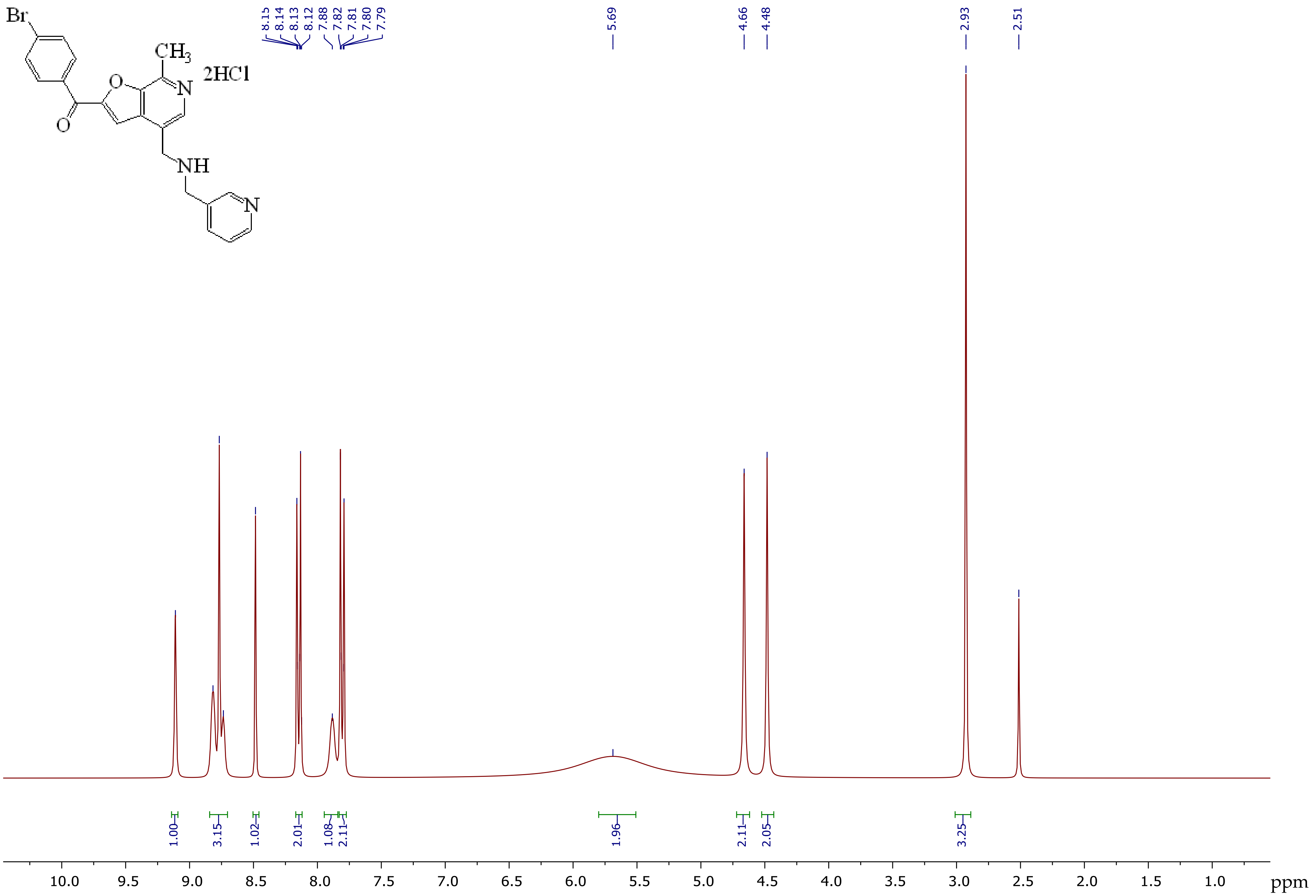

(1,4-Dihydrobenzo[d][1,2]dioxin-6-yl)(4-(((3,4-dimethoxybenzyl)amino)methyl)-7-methylfuro[2,3-c]pyridin-2-yl)methanone hydrochloride (**PD12**).

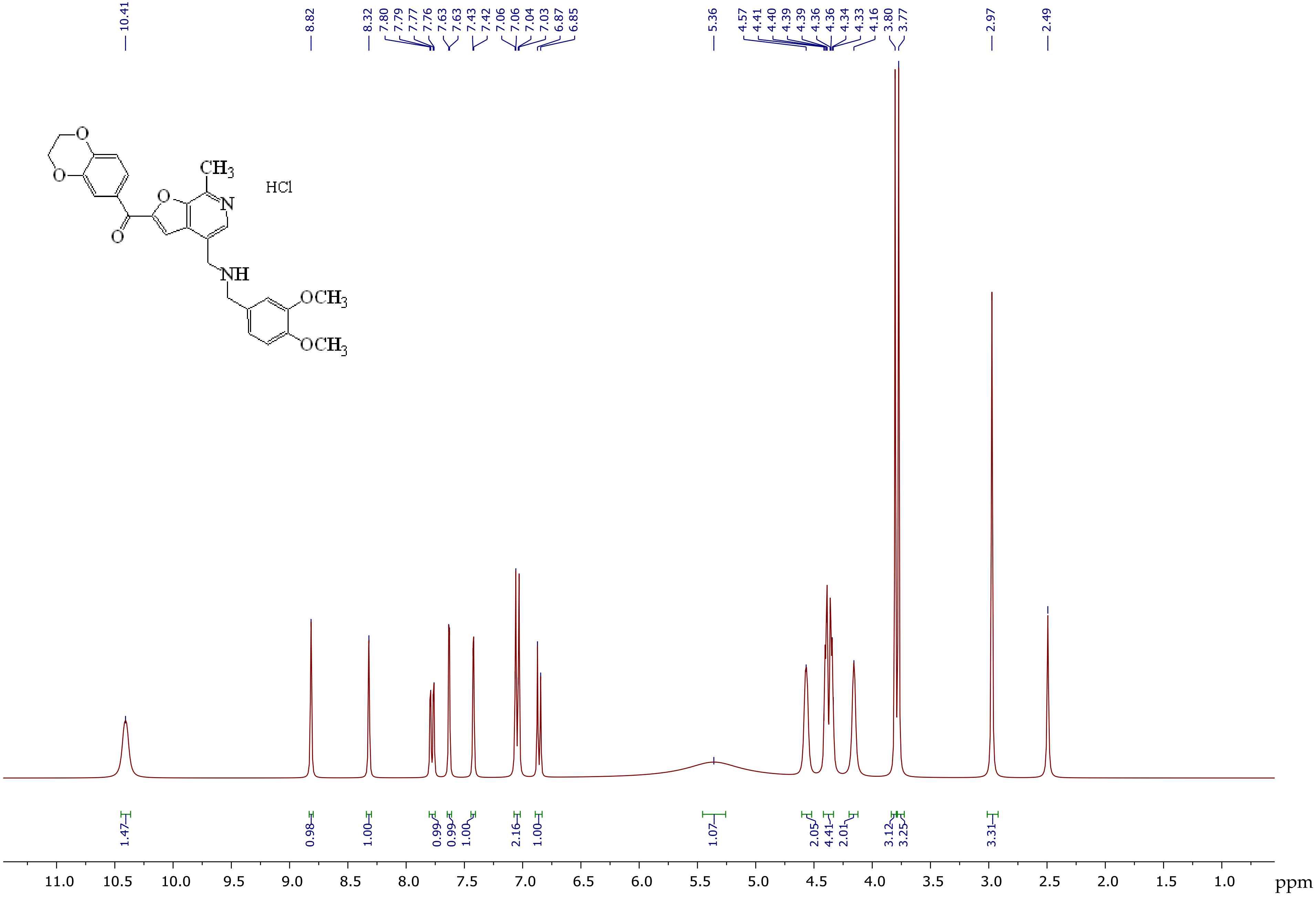

(1,4-Dihydrobenzo[d][1,2]dioxin-6-yl)(7-methyl-4-((4-(pyridin-3-ylmethyl)piperazin-1-yl)methyl)furo[2,3-c]pyridin-2-yl)methanone dihydrochloride (**PD13**).

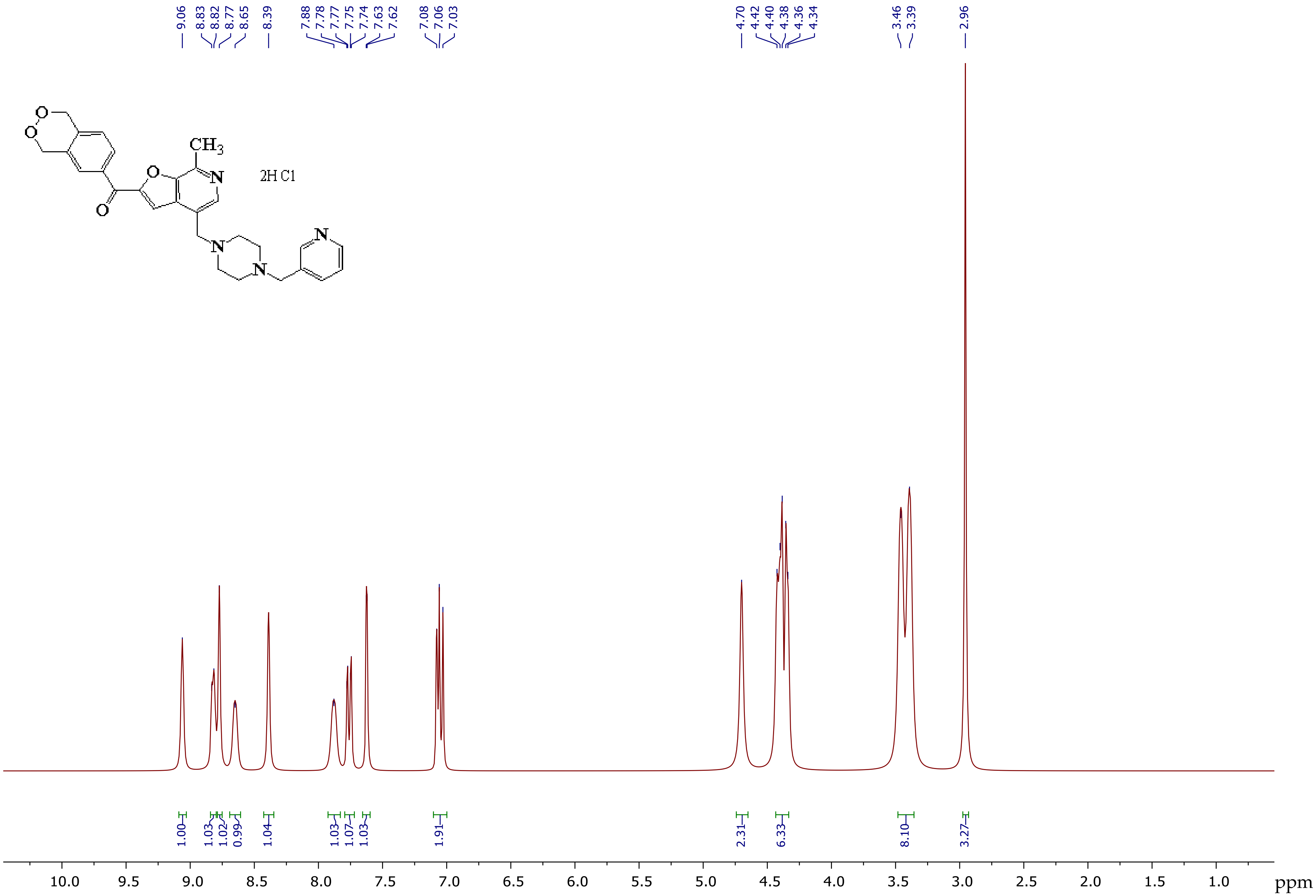

(1,4-Dihydrobenzo[d][1,2]dioxin-6-yl)(4-((4-(2-hydroxyethyl)piperazin-1-yl)methyl)-7-methylfuro[2,3-c]pyridin-2-yl)methanone dihydrochloride (**PD14**).

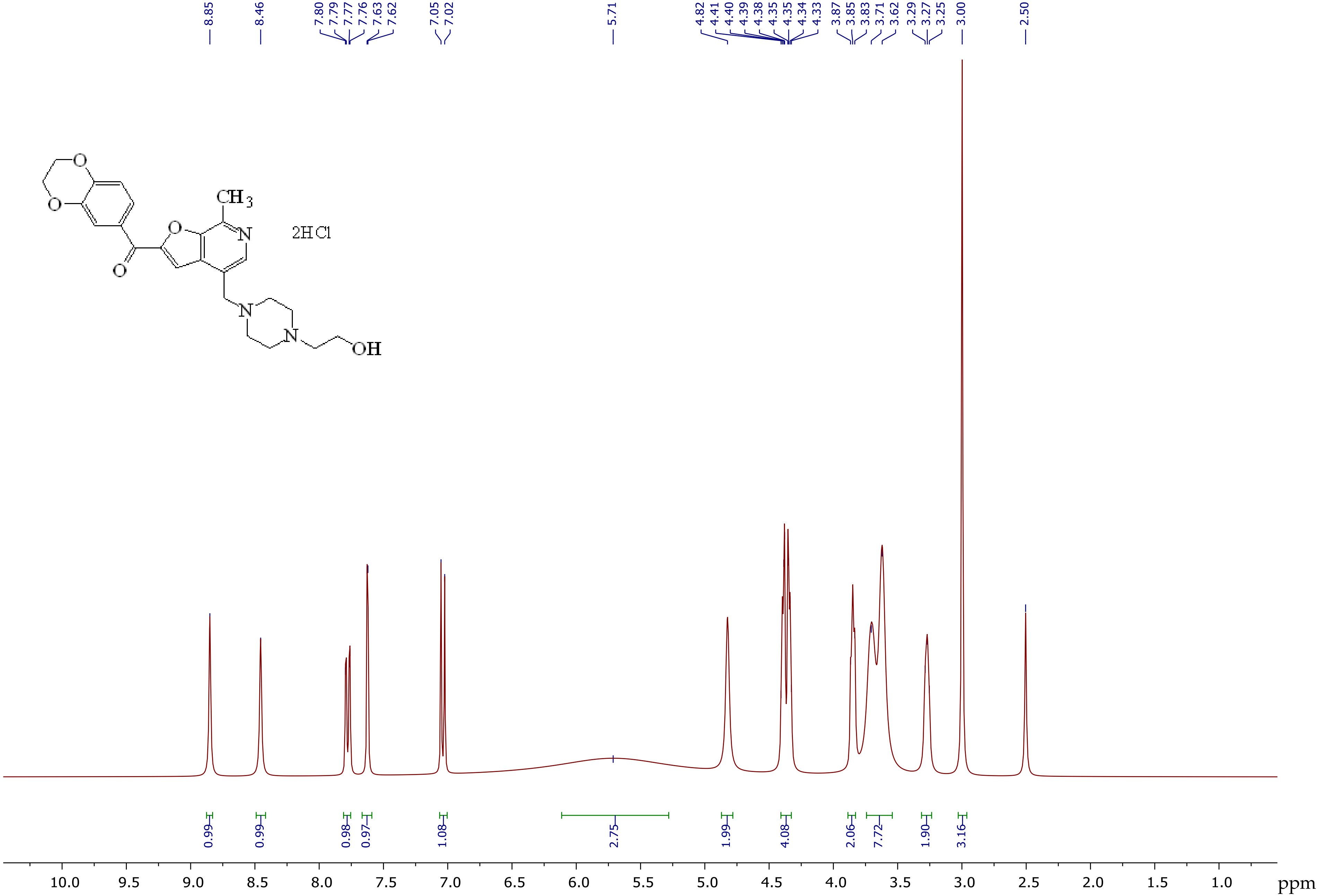

^{13}C NMR Spectra of compounds PD1-PD14.

[4-(Hydroxymethyl)-7-methylfuro[2,3-c]pyridin-2-yl](4-methoxyphenyl)methanone (**PD1**).

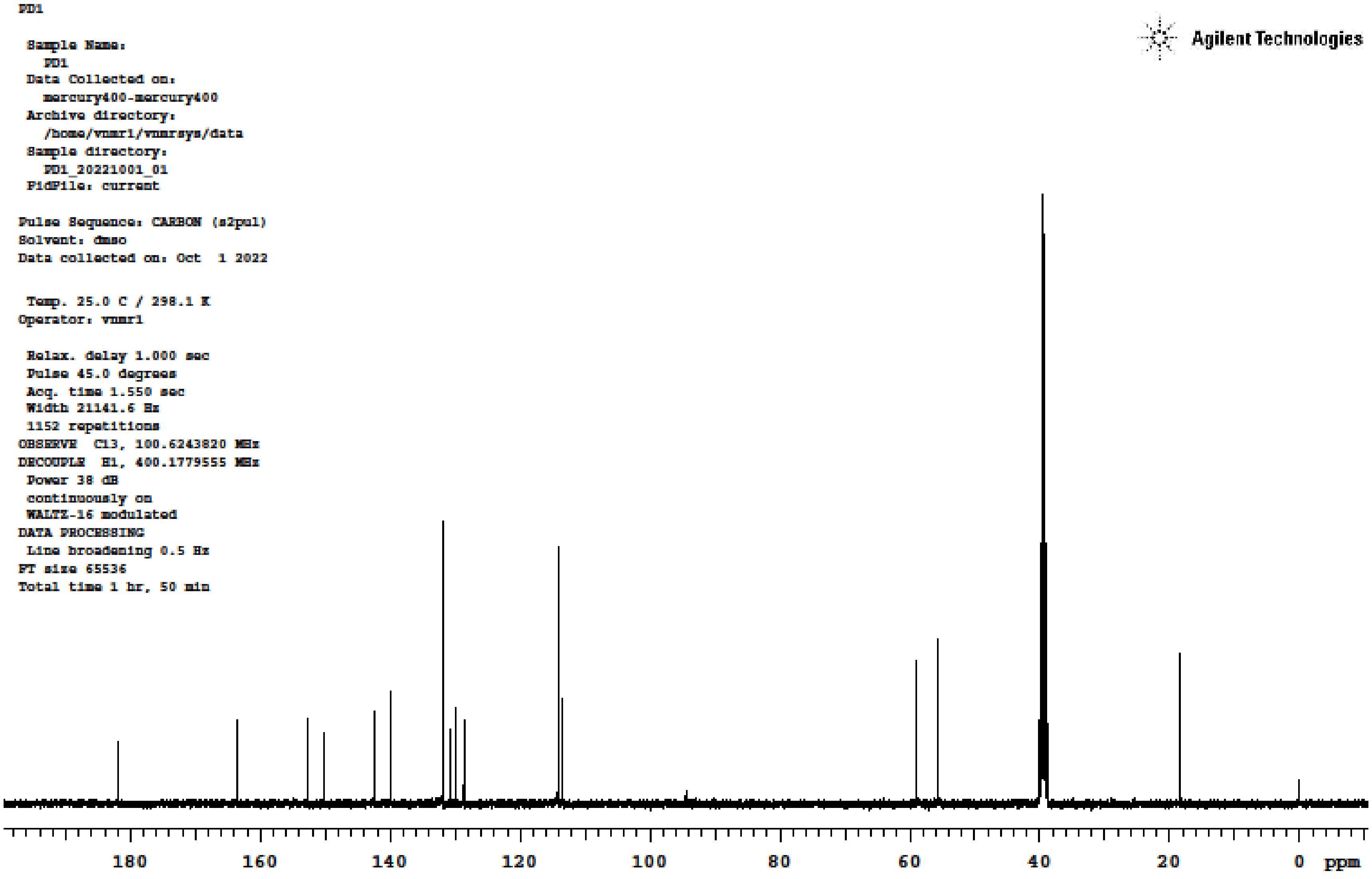

(1,4-Dihydrobenzo[d][1,2]dioxin-6-yl)(4-(hydroxymethyl)-7-methylfuro[2,3-c]pyridin-2-yl)methanone (**PD2**)

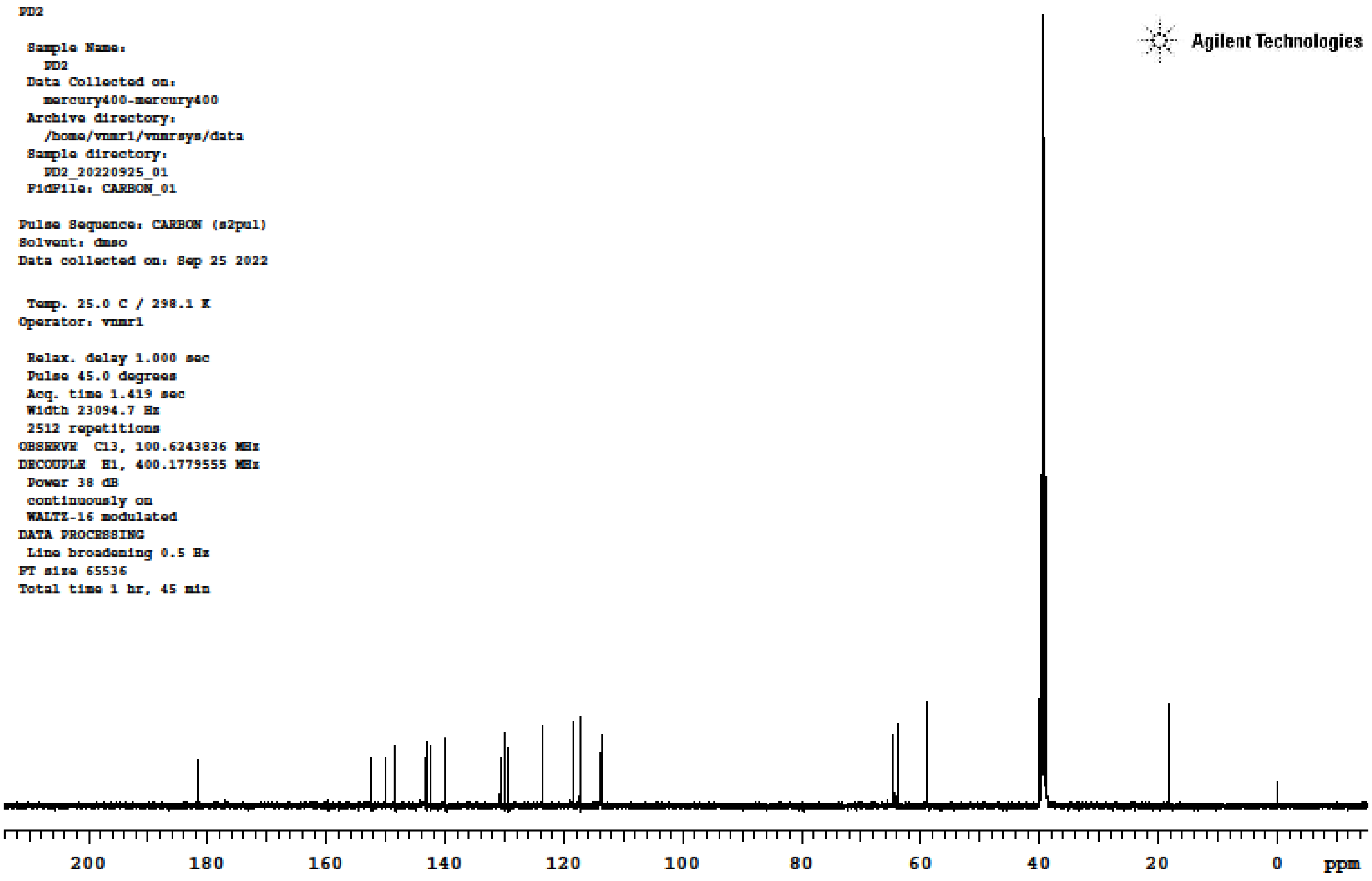

(4-Bromophenyl){7-methyl-4-[(4-(pyridin-2-ylmethyl)piperazin-1-yl]methyl}furo[2,3-c]pyridin-2-yl)methanone dihydrochloride (**PD3**).

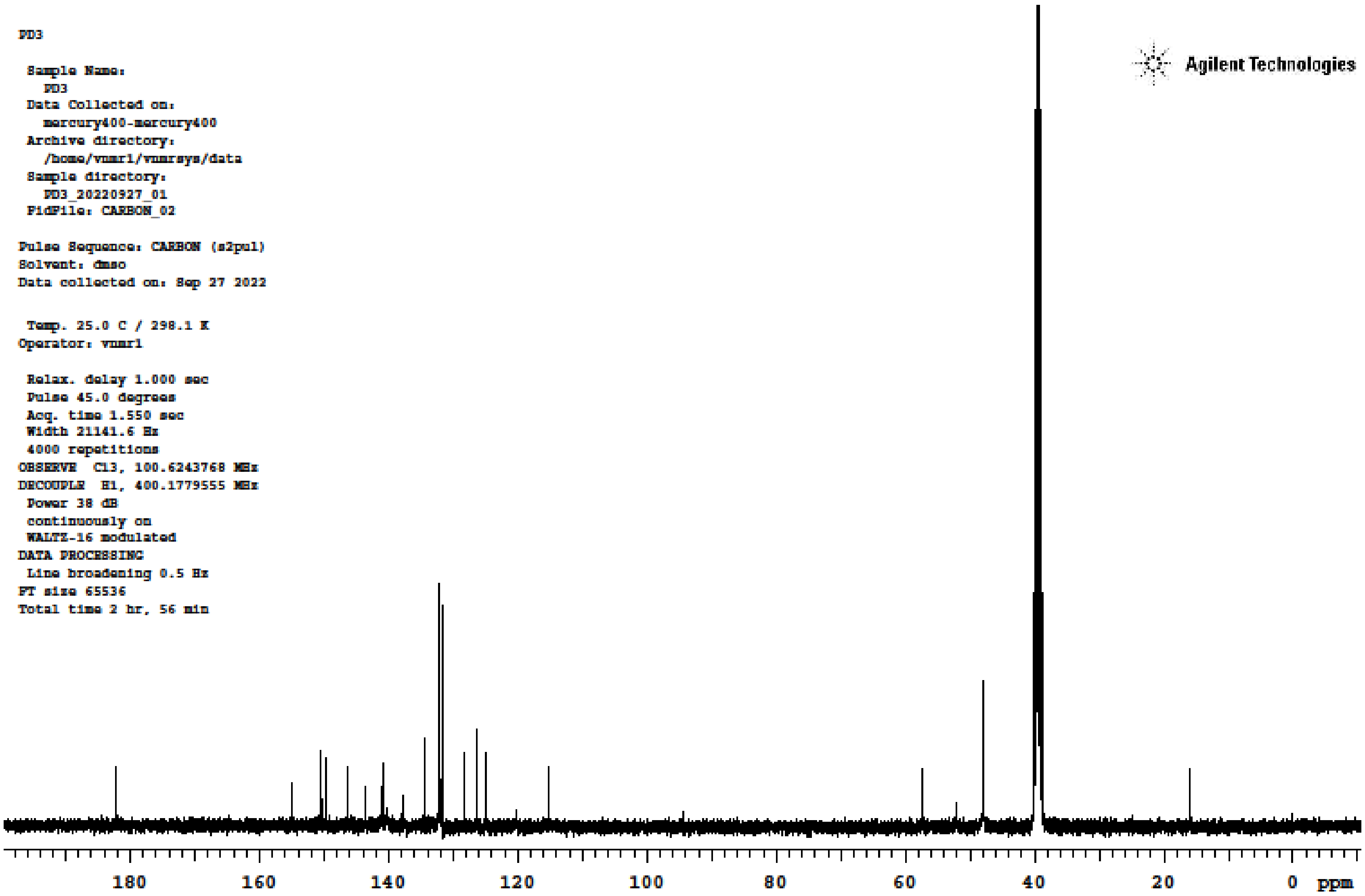

4-Bromophenyl)(7-methyl-4-((4-(pyridin-3-ylmethyl)piperazin-1-yl)methyl)furo[2,3-c]pyridin-2-yl)methanone (**PD4**).

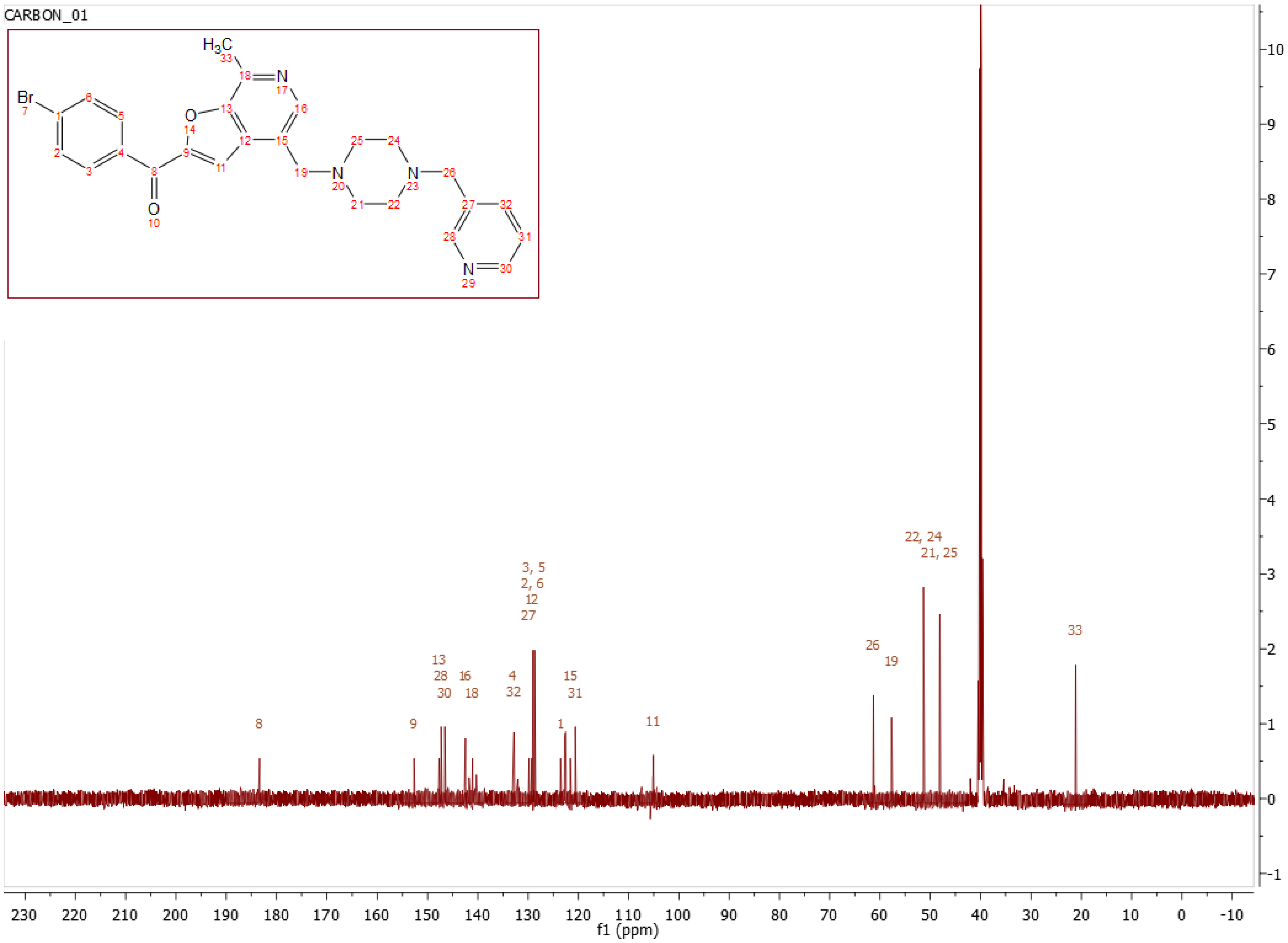

(4-((1H-Imidazol-1-yl)methyl)-7-methylfuro[2,3-c]pyridin-2-yl)(4-bromophenyl)methanone hydrochloride (**PD5**).

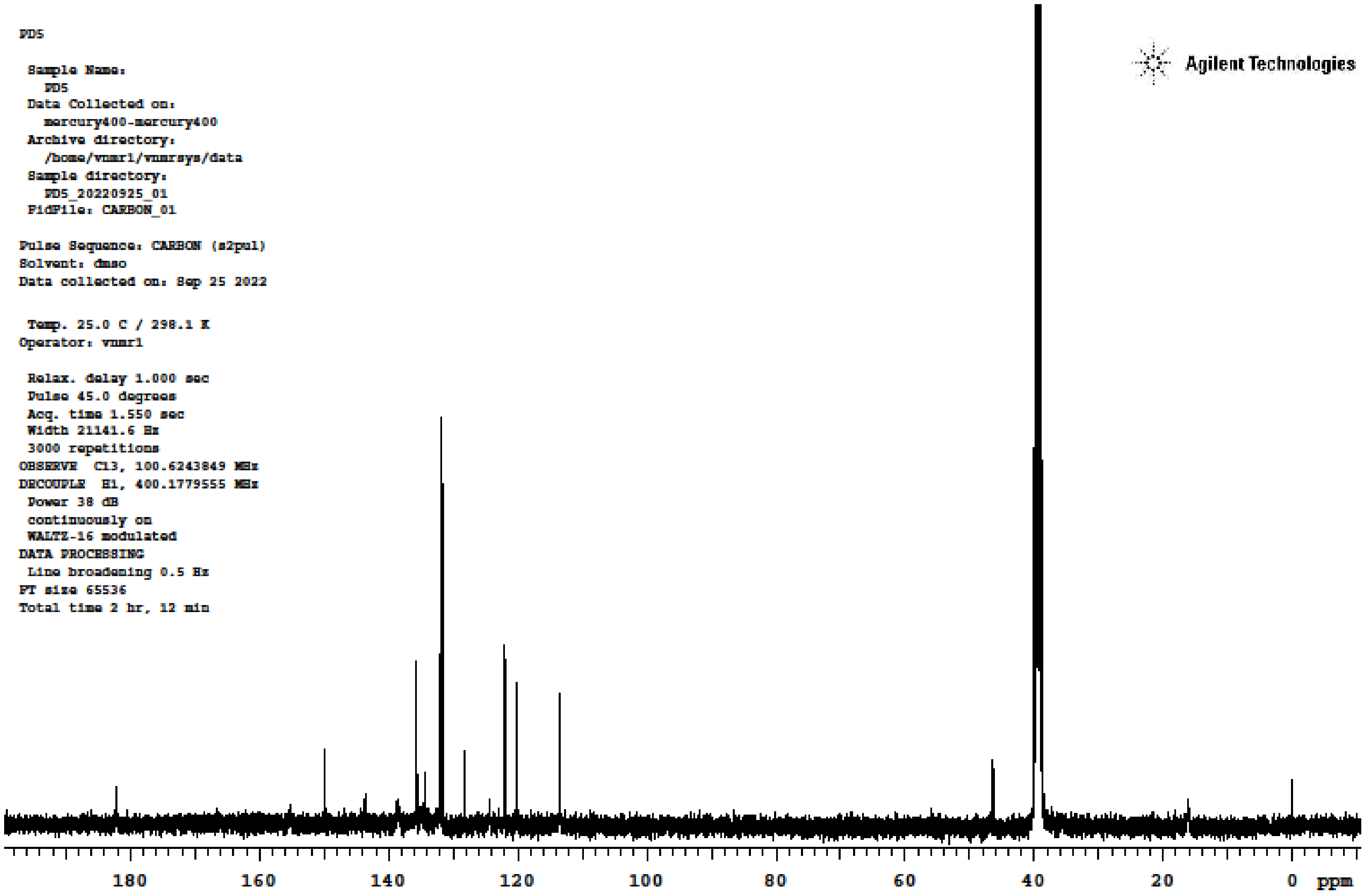

4-Bromophenyl)(4-((4-(2-hydroxyethyl)piperazin-1-yl)methyl)-7-methylfuro[2,3-c]pyridin-2-yl)methanone (**PD6**).

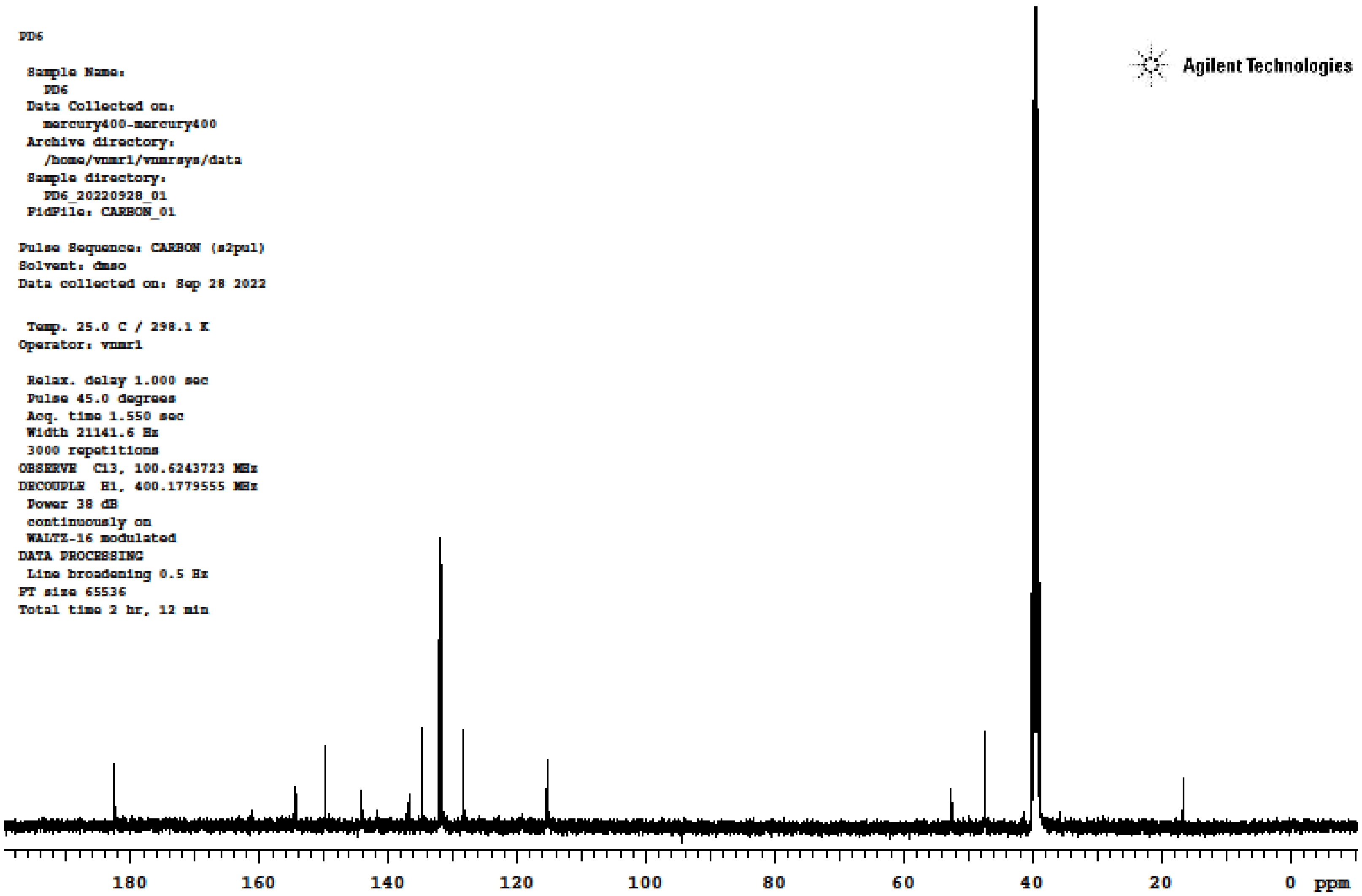

(4-Bromophenyl)(4-((4,5-dichloro-1H-imidazol-1-yl)methyl)-7-methylfuro[2,3-c]pyridin-2-yl)methanone (**PD7**).

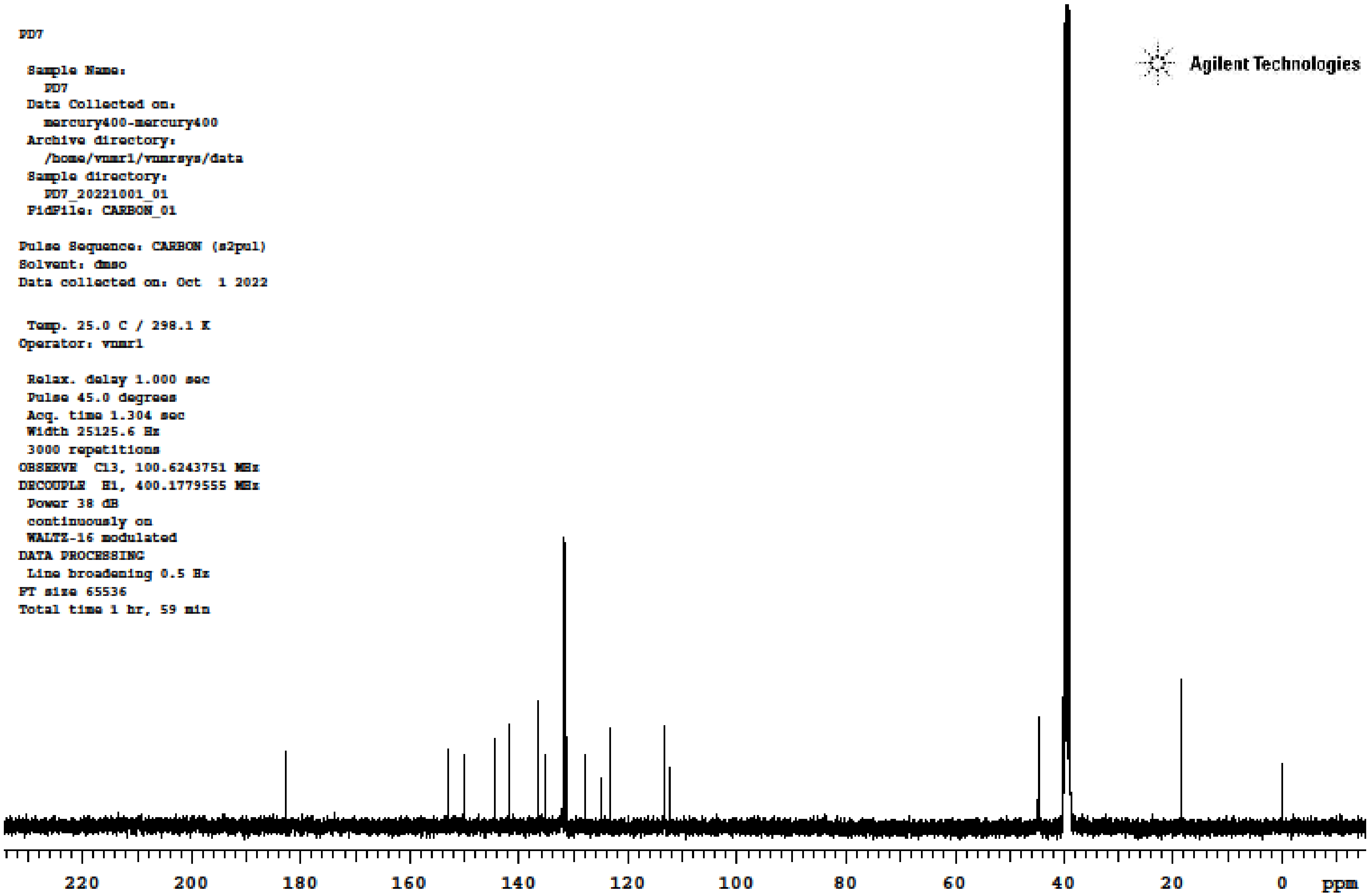

(4-Bromophenyl)(4-((3,4-dihydroisoquinolin-2(1H)-yl)methyl)-7-methylfuro[2,3-c]pyridin-2-yl)methanone (**PD8**).

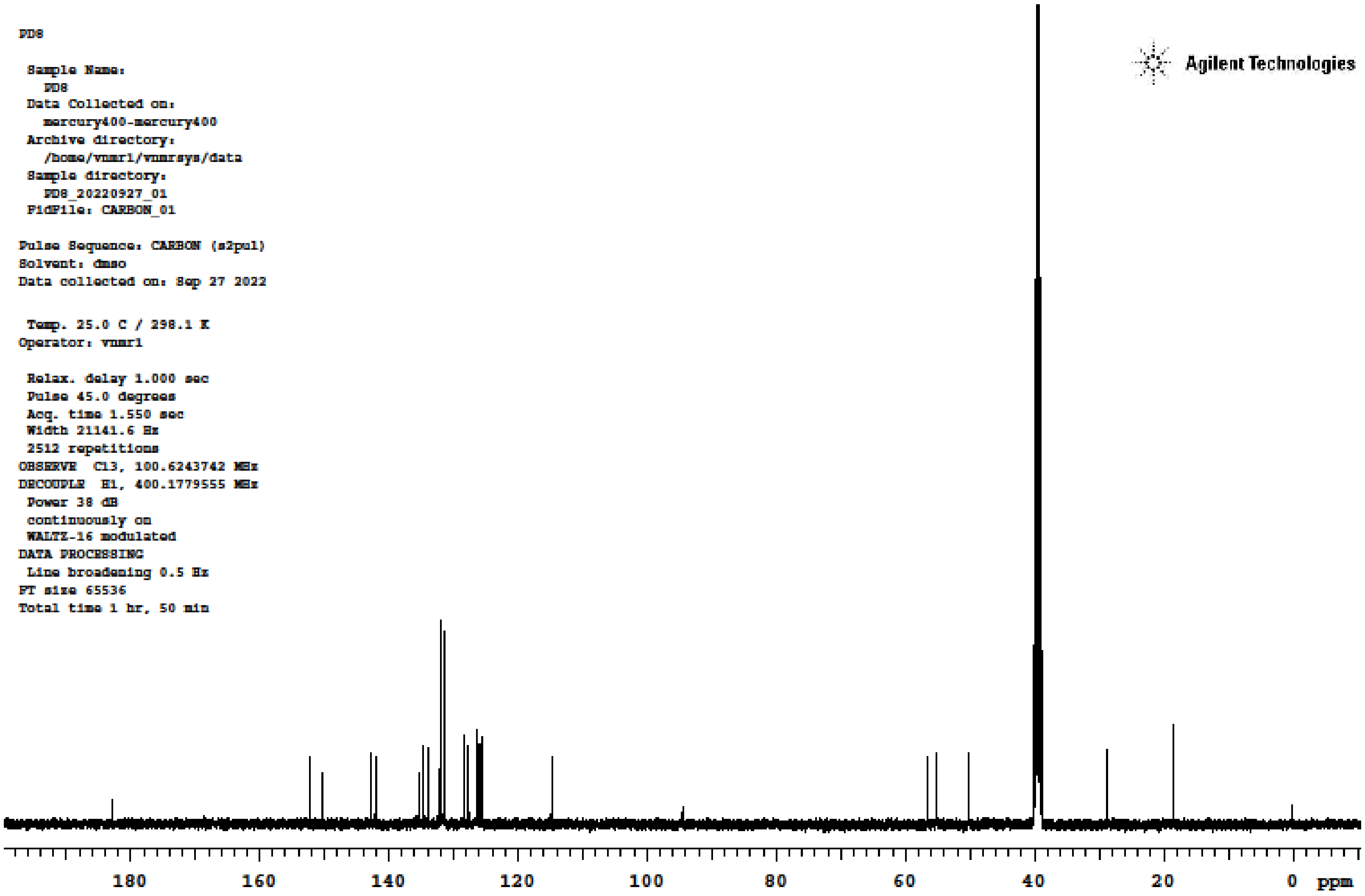

3-((2-(4-Bromobenzoyl)-7-methylfuro[2,3-c]pyridin-4-yl)methyl)piperazine-1-carbaldehyde hydrochloride (**PD9**).

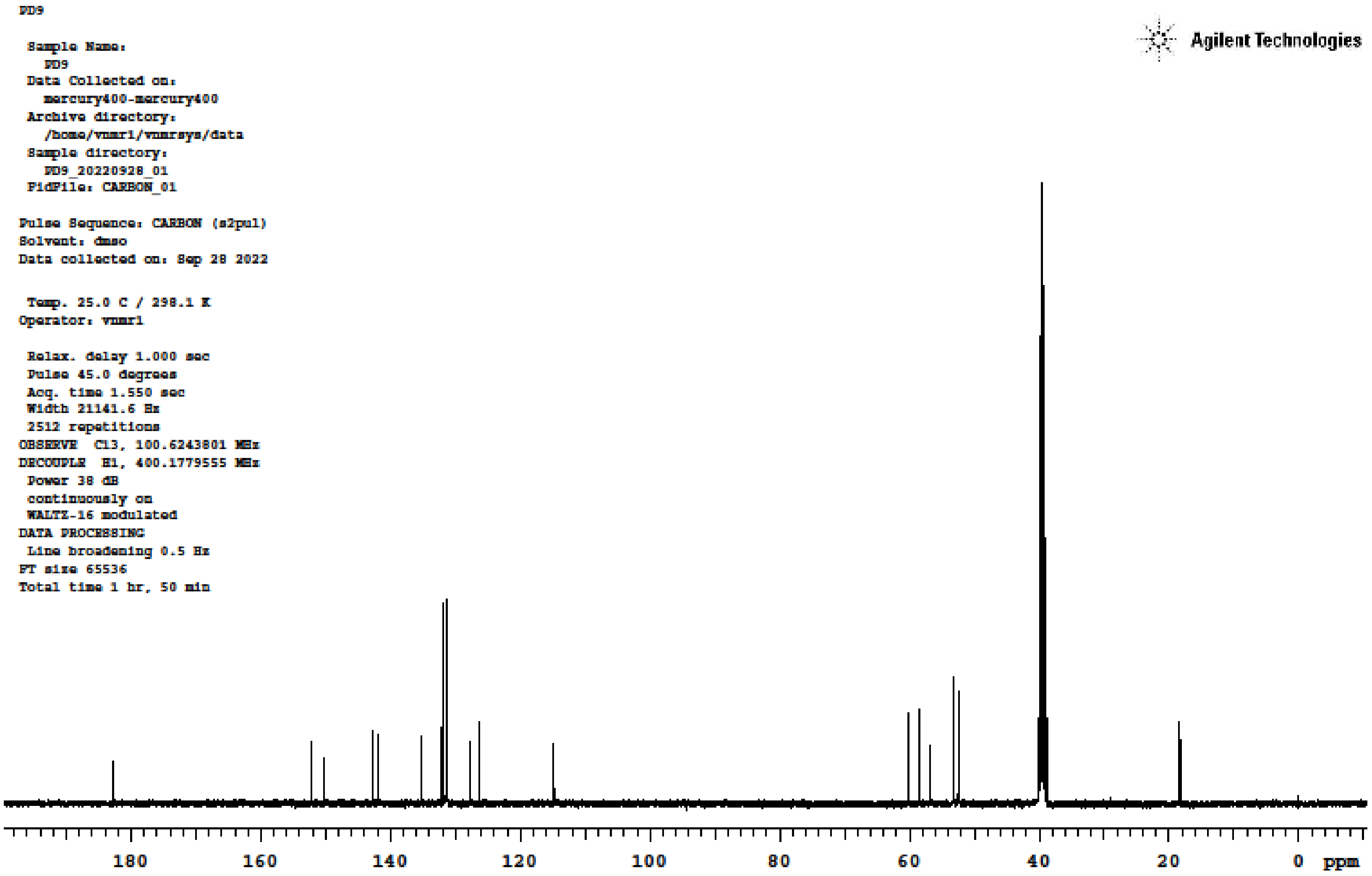

3-(4-((2-(4-Bromobenzoyl)-7-methylfuro[2,3-c]pyridin-4-yl)methyl)piperazin-1-yl)propanenitrile (**PD10**).

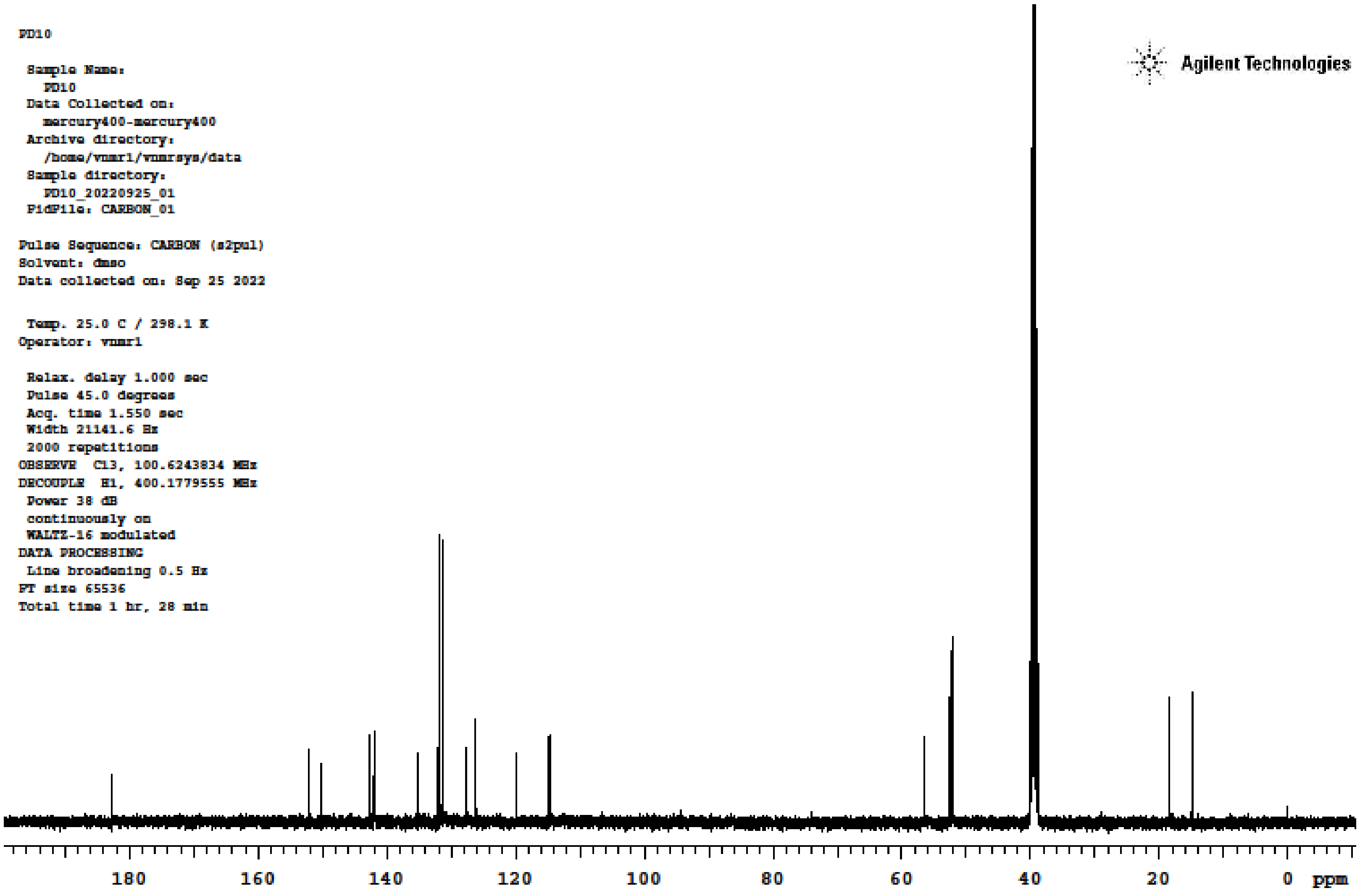

(4-Bromophenyl)(7-methyl-4-(((pyridin-3-ylmethyl)amino)methyl)furo[2,3-c]pyridin-2-yl)methanone dihydrochloride (**PD11**).

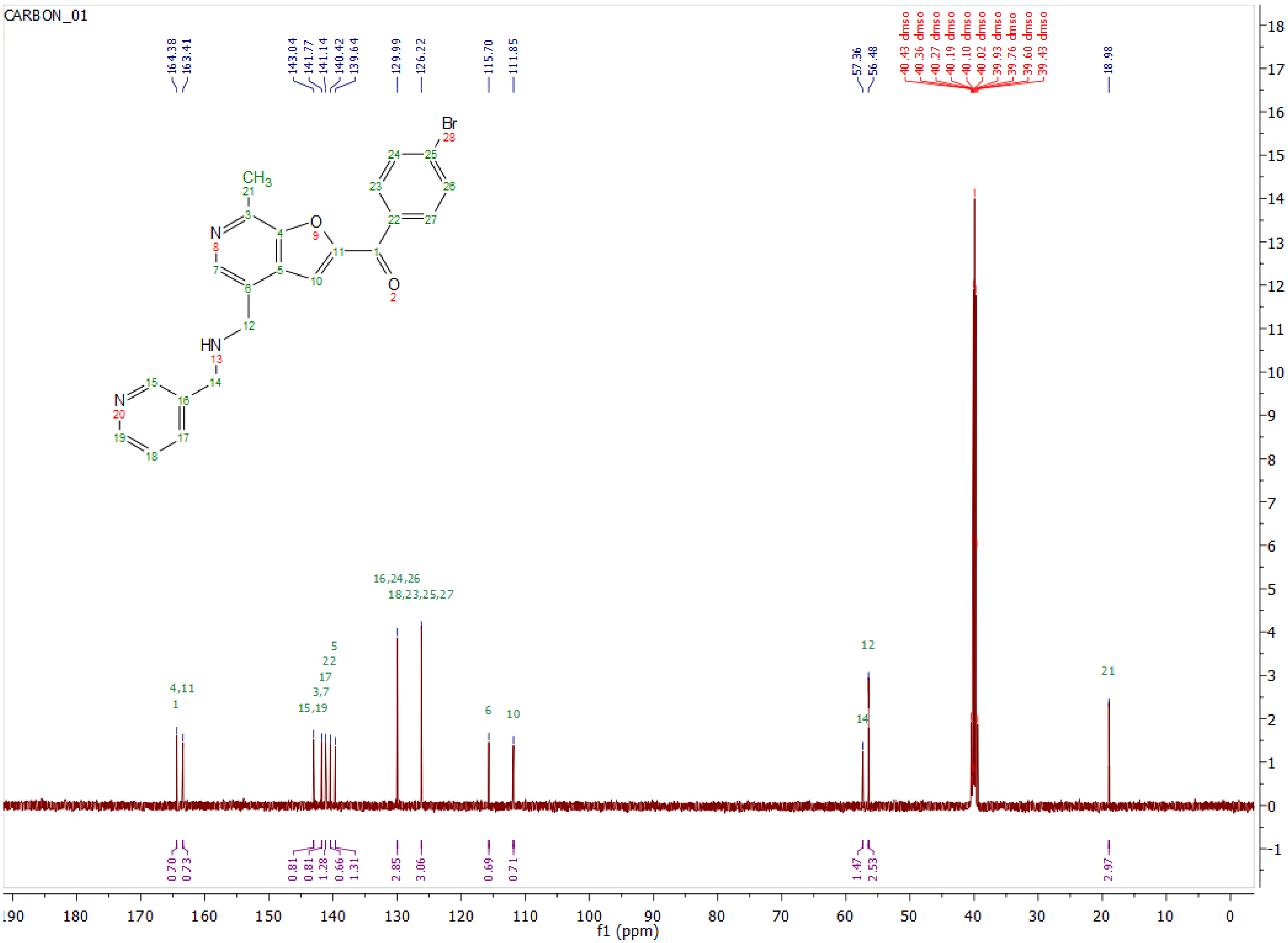

(1,4-Dihydrobenzo[d][1,2]dioxin-6-yl)(4-(((3,4-dimethoxybenzyl)amino)methyl)-7-methylfuro[2,3-c]pyridin-2-yl)methanone hydrochloride (**PD12**).

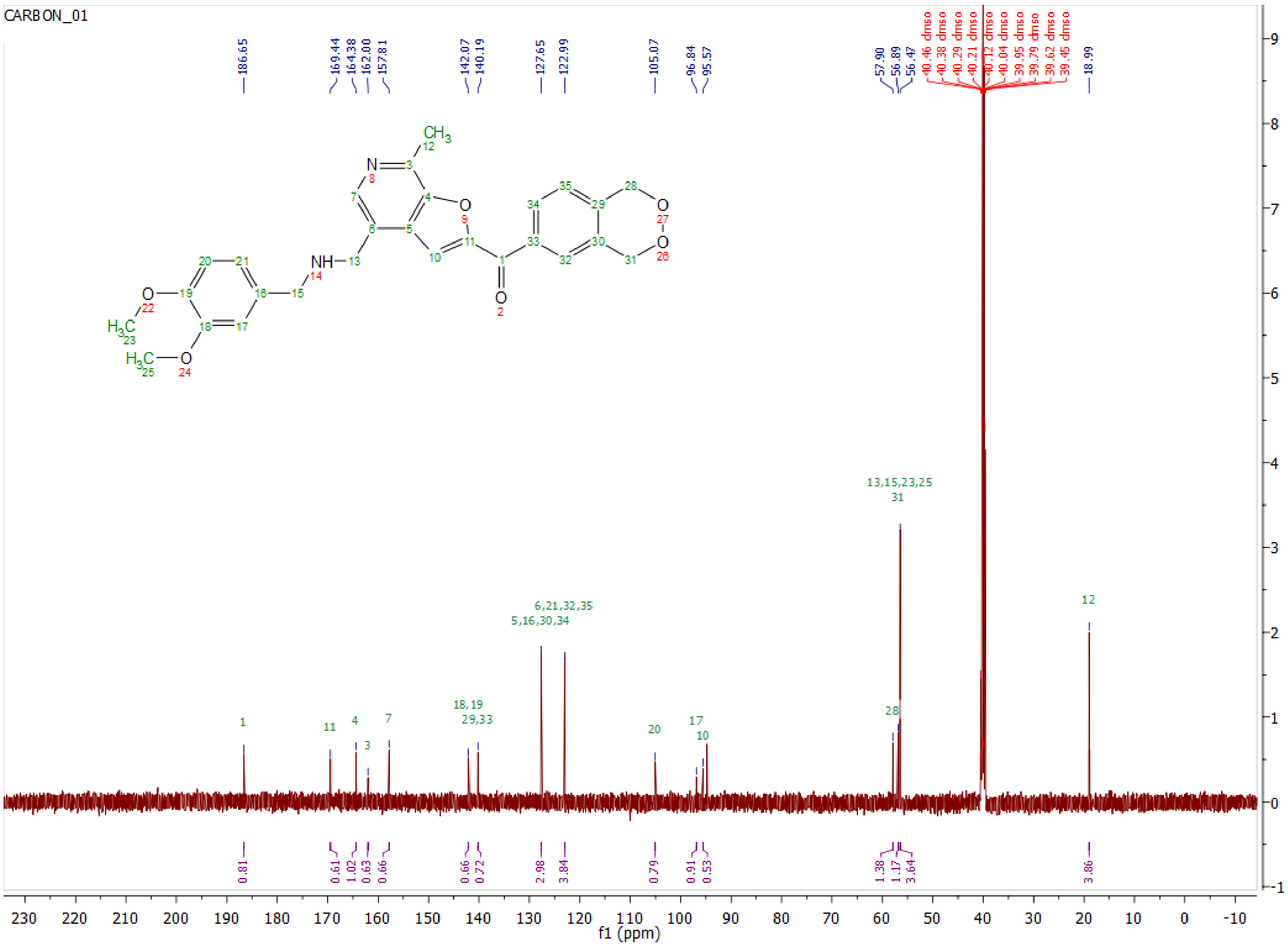

1,4-Dihydrobenzo[d][1,2]dioxin-6-yl)(7-methyl-4-((4-(pyridin-3-ylmethyl)piperazin-1-yl)methyl)furo[2,3-c]pyridin-2-yl)methanone dihydrochloride (**PD13).**

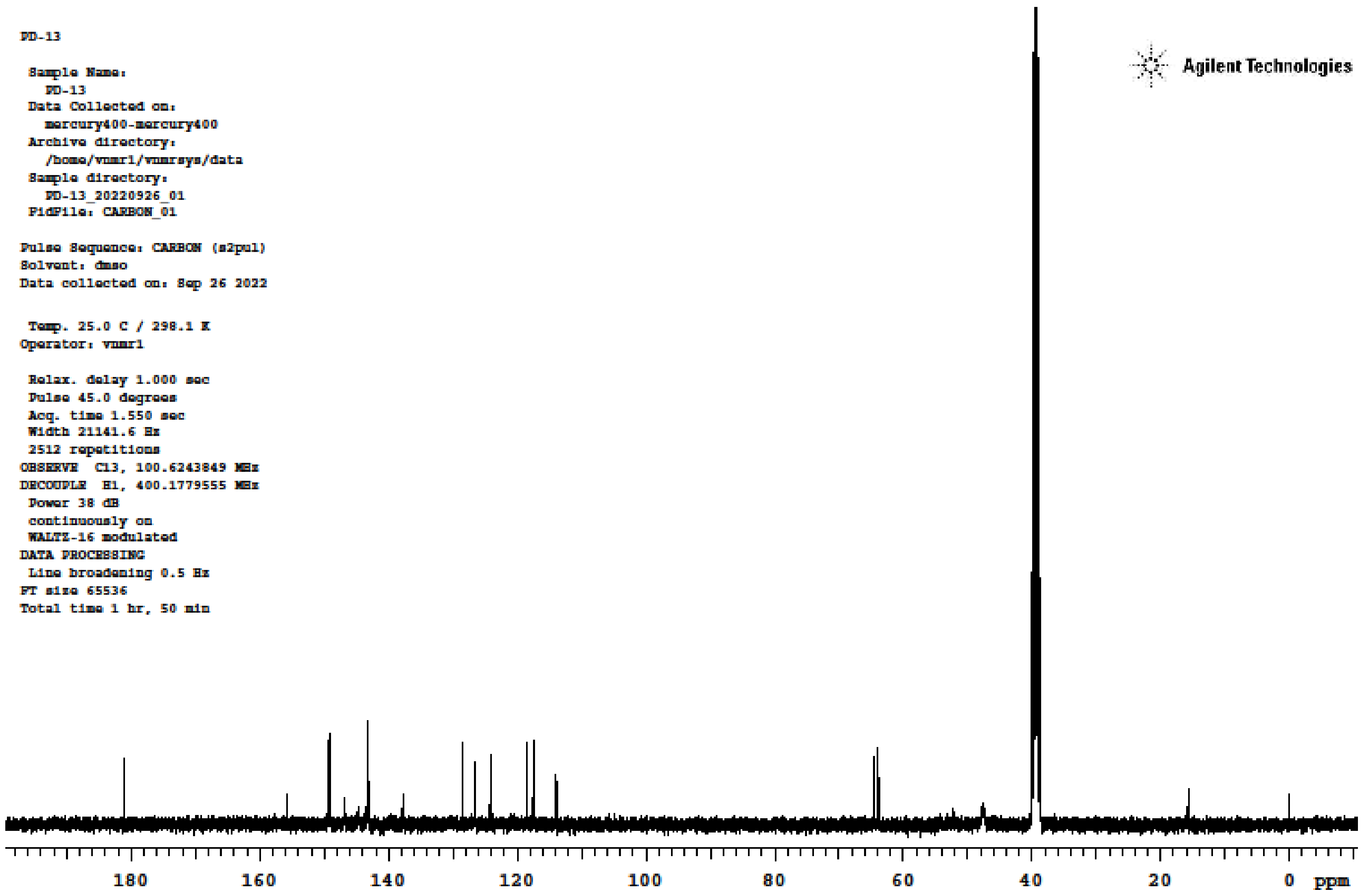

(1,4-Dihydrobenzo[d][1,2]dioxin-6-yl)(4-((4-(2-hydroxyethyl)piperazin-1-yl)methyl)-7-methylfuro[2,3-c]pyridin-2-yl)methanone dihydrochloride (**PD14**).

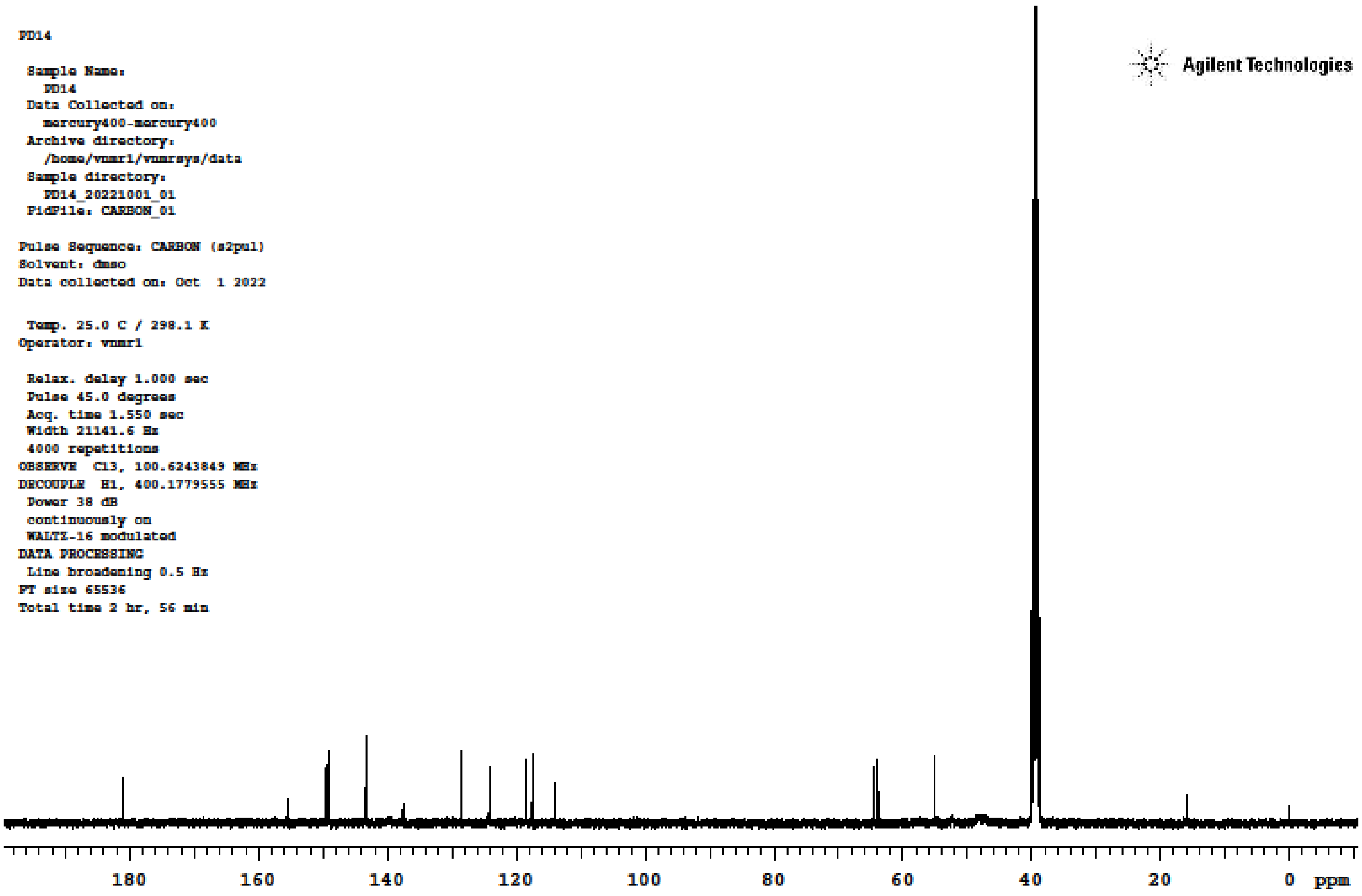

Supplement: Supplementary file 1 [file molecules-28-03014-s001.zip › Supplementary/Electronic supplementary materials Molecules LAST.pdf]
